# RNA Virus Diversity in Birds and Small Mammals From Qinghai–Tibet Plateau of China

**DOI:** 10.3389/fmicb.2022.780651

**Published:** 2022-02-18

**Authors:** Wentao Zhu, Jing Yang, Shan Lu, Dong Jin, Ji Pu, Shusheng Wu, Xue-Lian Luo, Liyun Liu, Zhenjun Li, Jianguo Xu

**Affiliations:** ^1^State Key Laboratory of Infectious Disease Prevention and Control, Chinese Center for Disease Control and Prevention, National Institute for Communicable Disease Control and Prevention, Beijing, China; ^2^Shanghai Public Health Clinical Center, Fudan University, Shanghai, China; ^3^Research Units of Discovery of Unknown Bacteria and Function, Chinese Academy of Medical Sciences, Beijing, China; ^4^Yushu Prefecture Center for Disease Control and Prevention, Yushu, China; ^5^Research Institute of Public Heath, Nankai University, Tianjin, China

**Keywords:** virome, bird, rodent, small mammals, Qinghai–Tibet Plateau, diversity, fecal sample

## Abstract

Most emerging and re-emerging viruses causing infectious diseases in humans and domestic animals have originated from wildlife. However, current knowledge of the spectrum of RNA viruses in the Qinghai-Tibet Plateau in China is still limited. Here, we performed metatranscriptomic sequencing on fecal samples from 56 birds and 91 small mammals in Tibet and Qinghai Provinces, China, to delineate their viromes and focused on vertebrate RNA viruses. A total of 184 nearly complete genome RNA viruses belonging to 28 families were identified. Among these, 173 new viruses shared <90% amino acid identity with previously known viral sequences. Several of these viruses, such as those belonging to genera *Orthonairovirus* and *Hepatovirus*, could be zoonotic viruses. In addition, host taxonomy and geographical location of these viruses showed new hosts and distribution of several previously discovered viruses. Moreover, 12 invertebrate RNA viruses were identified with <40% amino acid identity to known viruses, indicating that they belong to potentially new taxa. The detection and characterization of RNA viruses from wildlife will broaden our knowledge of virus biodiversity and possible viral diseases in the Qinghai–Tibet Plateau.

## Introduction

The majority of emerging and re-emerging viral infectious diseases in humans have originated from wildlife, including rodents and birds, and are increasing with time (Jones et al., 2008; Wu et al., 2017; He et al., 2021). The severe acute respiratory syndrome coronavirus 2 (SARS-CoV-2) pandemic has reminded us of the pathogenetic potential of viruses and their capacity to cause deadly outbreaks on a global scale. RNA viruses are likely to be present in all cellular life (Koonin et al., 2006) and constitute the vast majority of the global virome (Mu et al., 2017; Wolf et al., 2018; Kondo et al., 2020). Most studies primarily focus on identifying RNA viruses that are pathogenic to humans and animals. However, emerging viruses appear to be well adapted to thrive in their reservoir host with little or no obvious evidence of clinical features (Wu et al., 2018). Besides, our knowledge of the viral population and its ecological diversity harbored by wildlife is largely obscure (Paez-Espino et al., 2016; Carroll et al., 2018; Wu et al., 2018). Therefore, an in-depth understanding of the spectrum of viruses existing in wildlife, in addition to their prevalence and distribution, will contribute to the prevention and control of emerging viral infectious diseases of wildlife origin (Olival et al., 2017).

More than 10,000 living species of birds, the richest lineage of extant tetrapod vertebrates, are distributed all over the world and show a broad diversity in morphology, and ecology (Gill et al., 2015; Prum et al., 2015). Birds exhibit a flocking behavior and can fly over great distances, thereby effectively spreading emerging and re-emerging RNA viruses, such as avian influenza viruses, Usutu virus, West Nile virus, and coronaviruses, among themselves and to humans and other animals, Reed et al. (2003), Olsen et al. (2006), Woo et al. (2012), Lühken et al. (2017), Zhu et al. (2021c). However, studies on avian viruses have mainly focused on influenza viruses, and little attention has been given to virus biodiversity.

Small mammals, such as shrews, rats, hamsters, and pikas, vary widely in preferred food, habits, habitat use, and lifestyle (Animal and Veterinary Service, 2021). Rodentia is the most diverse order of class Mammalia. It includes 33 families with more than 2,200 species and accounts for about 43% of all mammalian species (Blanga-Kanfi et al., 2009). Rodents are hosts and reservoirs of several important emerging and re-emerging zoonotic viruses, such as Rift Valley fever virus, bornavirus, Lassa virus, tick-borne encephalitis virus, lymphocytic choriomeningitis virus, and hantaviruses, which cause severe diseases in humans (Woo et al., 2012; Rabiee et al., 2018). The transmission and prevalence of rodent-borne viral diseases vary in different regions, and some of these viruses exhibit a global distribution pattern (Mills, 2005; Rabiee et al., 2018). Rodents live in close proximity with humans and play an important role in the interaction between human and arthropod vectors and other wildlife (Blanga-Kanfi et al., 2009; Meerburg et al., 2009). Further studies of the viral spectrum in the wild rodent populations could help in understanding viral evolution, emergence, and biodiversity.

The Qinghai–Tibet Plateau is a global biodiversity hotspot with diverse geographical and topographic characteristics. It is the highest and largest plateau (average elevation >4,500 m) in the world with expansive planation surfaces, mountain ranges, and basins (Zhou et al., 2006).

Knowledge of virus biodiversity is limited, and zoonotic viruses are still poorly understood. Our preliminary exploration of pikas and marmots (Luo et al., 2018; Zhu et al., 2021b) was used to establish the baseline of RNA viruses in the Qinghai–Tibet plateau. Here, we report RNA viruses from birds and small mammals in Tibet and Qinghai Provinces, China, to outline their viral spectrum, including evolutionary, genetic, and distributional characteristics.

## Materials and Methods

### Sample Collection

In April 2018, fecal samples of 31 birds and 41 rats were collected from various locations in the Cona County, Tibet Province (4,360 m above sea level; Supplementary Figure 1 and Supplementary Table 1). In July 2019, fecal samples of 25 birds and 50 small mammals were collected from various locations in Yushu and Nangqian Counties of Qinghai Province (3,890 m above sea level; Supplementary Figure 1 and Supplementary Table 1). Small mammals were captured in their natural habitat using mousetraps, and birds were coincidentally captured while catching pikas near pika holes (Zhu et al., 2021b). The wild animals were euthanized and dissected. Their fecal samples were collected and preserved in maintenance medium consisting of Hank’s balanced salt solution (pH 7.4) with penicillin G (100 U/ml) and streptomycin (50 μg/ml), and kept at −20°C while transfer to the laboratory, and stored at −80°C in the laboratory. The species of animals were identified using the mitochondrial cytochrome b (*Cyt b*) gene (Sorenson et al., 1999) or by morphological observation by experts. The sampling process was conducted by the local center for disease control and prevention (CDC) as part of the National Surveillance Program for Plague in Wildlife, and authorized by the Ethics Committee of National Institute for Communicable Disease Control and Prevention, China CDC (ICDC-2019012).

### RNA Extraction

The process was mainly followed as previously reported (Wu et al., 2018). Briefly, each specimen was homogenized in phosphate buffer saline (PBS). Clear suspensions were obtained by centrifugation at 15,000 × *g* for 20 min and were filtered using a 0.22-μm polyvinylidene difluoride filter. The filtered supernatant was centrifuged at 300,000 × *g* for 2 h at 4°C. Pellets were re-suspended in PBS and digested using the RNase-Free DNase I Kit (Qiagen) at 37°C for 1 h. RNA was extracted using the QIAamp Viral RNA Mini Kit (Qiagen). RNA concentration and quality of each sample were determined using Qubit (Thermo Fisher) and 2100 Bioanalyzer (Agilent). The same animal classes from the same sampling county were divided into the same group (Supplementary Table 1), resulting in four groups. RNA of each sample of the same group was pooled in equal quantity.

### RNA Library Construction and Next-Generation Sequencing

To facilitate virus discovery, rRNA of each library was removed as previously descripted (Zhu et al., 2021a) using the Ribo-Zero Gold rRNA Removal Kit (Illumina). Libraries were constructed using the TruSeq Stranded Total RNA Library Prep Gold Kit (Illumina) according to manufacturer’s instructions. RNA was fragmented, and random hexamers were used to transcribe RNA into cDNA. The second strand of cDNA was obtained using the DNA polymerase I large fragment. The next steps included end repair, adapter ligation, purification, and fragment selection. The constructed libraries were sequenced using the Illumina HiSeq 2000 platform with 150 bp paired-end reads.

### Virus Discovery

RNA viruses were detected according to an established metatranscriptomic pipeline (Shi et al., 2018). Raw reads were trimmed to remove the adapter and low-quality reads (<Q20) using Trimmomatic v0.32 (Bolger et al., 2014). The obtained high-quality reads were assembled *de novo* per pool using both Trinity v2.4.0 (Grabherr et al., 2011) and Megahit v1.1.2 (Li et al., 2015). The resulting contigs were first annotated using the database including all reference virus proteins downloaded from NCBI^1^ by Diamond BLASTx with the e-values to 1e^–5^ (Buchfink et al., 2015). In an attempt to identify highly divergent viral sequences, the assembled contigs were BLAST searched against the conserved domain database (CDD) v3.14 (Lu et al., 2020). Subsequently, the obtained viral contigs were verified and BLAST searched against both non-redundant protein and nucleotide databases, and contigs showing similarity to the host, plant, bacterial, and fungal sequences were eliminated. The resulting viral contigs were compared with their closely related members, and those with all viral proteins of the corresponding genus or family were retained for further analyses. The RNA-Seq by expectation–maximization algorithm (Li and Dewey, 2011) was used to quantify the abundance of contigs. Finally, viral contigs sharing <90% RNA-dependent RNA polymerase (RdRp) amino acid identity with any previously known virus were identified as new viruses (Shi et al., 2016).

### Genomic and Phylogenetic Analyses

Putative open reading frames (ORFs) of viral genomes were predicted using NCBI ORF Finder.^2^ The putative function of viral protein was annotated using CDD.^3^ Genetic distance (p-dist) was estimated using MEGA X software (Kumar et al., 2018), and amino acid identity was calculated using BioAider v1.314 (Zhou et al., 2020). Codon usage preferences were estimated using the codon usage similarity index (COUSIN^4^).

Potential viral contigs and their closely related members were aligned by multiple alignment program using MAFFT v7 (Katoh and Standley, 2013). The best-fit substitution models were estimated using ModelFinder in IQ-TREE v2 (Minh et al., 2020). Phylogenetic trees were constructed using PhyML v3.0 based on the maximum-likelihood method (Guindon et al., 2010) with corresponding substitution models from ModelFinder and 1,000 bootstrap replicates. Trees were finally edited and visualized in interactive Tree of Life v1.0 (Letunic and Bork, 2016).

### Inferring Zoonotic Potential

The probability of being able to infect humans was ranked using machine learning models with a 0.303 of the cutoff value following a recent report (Mollentze et al., 2021). Briefly, viral genomes were merged into a file in the FASTA sequence format, and the PredictNovel.R script was run.

### Confirmation and Prevalence Screening

Gaps between viral contigs with unassembled overlaps were filled by RT-PCR, which was performed using the PrimeScript™ One-Step RT-PCR Kit with specific primers (Supplementary Table 2) based on assembled sequences, and Sanger DNA sequencing. To confirm the assembly results, reads were mapped back to the viral sequences and aligned using Bowtie 2 (Langmead and Salzberg, 2012). To exclude the contigs belonging to expressed endogenous virus elements (EVEs), DNA was extracted from the corresponding samples and used for PCR amplification (Supplementary Table 2) using *Taq* DNA polymerase (TaKaRa). The sequence was eliminated if the PCR results were positive. In addition, these vial sequence (including host hits) were removed when performing BLASTn searches against the non-redundant nucleotide database.

To confirm the prevalence of vertebrate viruses, we designed specific primers (Supplementary Table 2) for viruses based on assembled genomes, and performed RT-PCR to screen corresponding viral sequence in individual samples. PCR products were subjected to gel purification and Sanger DNA sequencing. We did not submit the sequences from Sanger DNA sequencing to public databases.

## Results

Metatranscriptomic sequencing was performed on fecal samples collected from 31 birds (library XZNCD) and 41 rats (library XZSCD) from Tibet Province in April 2018, and 25 birds (library YSNCD) and 50 small mammals (library YSSCD) from Qinghai Province in July 2019. The samples were organized into four pools for high-throughput RNA sequencing according to animal species and sampling location (Supplementary Table 1). The four rRNA-depleted libraries resulted in 474,166,740 paired-end reads with 65,810,192–182,565,940 reads per pool (Supplementary Figure 2A), which were deposited in the NCBI Sequence Reads Archive under accession numbers SRR13847367, SRR13847389, SRR13847390, and SRR13857276.

### Virome Overview

**FIGURE 1:**
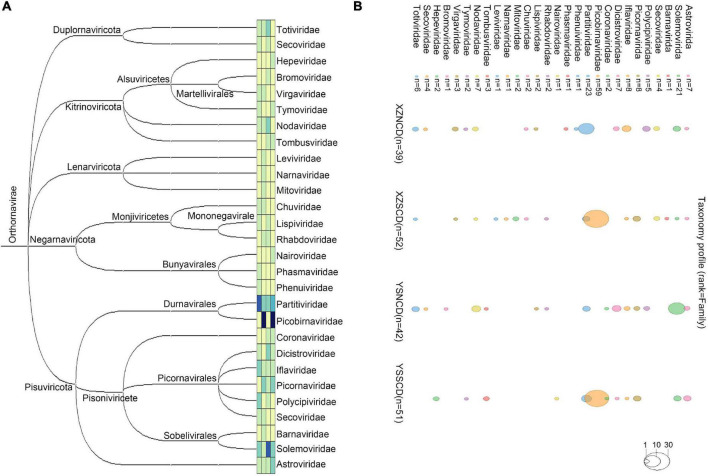
Overview of RNA viruses identified in this study. **(A)** Phylogenetic tree of the 28 virus families. The tree was constructed by loading the names of virus families and its corresponding numbers to STAMP v2 (Parks et al., 2014). **(B)** Bubble map showing the number of viruses identified in the corresponding family in each library. Same color squares and circles represent corresponding virus families before the squares. The total number of each family is labeled after corresponding squares. The viral number of corresponding families in each pool is indicated by circles of different sizes. The no-filled circles of three sizes represent 1, 10, 30 viruses, respectively.

A total of 184 complete or near-complete viral genomes that contain the complete RdRp domain were obtained (Figure 1 and Supplementary Table 3). Sequence comparisons indicated that 173 of them were divergent from previously known viruses, sharing <90% amino acid identity with known viruses. Coronaviruses detected in fecal samples have already been reported (Zhu et al., 2021c). Viruses of the family *Picobirnaviridae* accounted for >50% of the total number of viruses in the XZSCD and YSSCD libraries (Supplementary Figure 2B). All four libraries contained viruses from families *Astroviridae*, *Iflaviridae*, *Partitiviridae*, and *Solemoviridae* (Supplementary Figure 3).

To assess the amount of each viral RNA read, reads were mapped back to viral genomes. The viral family abundance among the four libraries showed marked differences (Supplementary Figure 2C). Viral families that revealed relatively high abundances were *Nodaviridae*, *Chuviridae*, *Partitiviridae*, *Dicistroviridae* and *Solemoviridae* in the XZNCD library; *Solemoviridae*, *Chuviridae*, *Picornaviridae* and *Mitoviridae* in the XZSCD library; *Totiviridae* in the YSNCD library; *Nairoviridae* in the YSSCD library (Supplementary Figure 2).

### Genomic Characterization and Phylogenetic Analysis of Vertebrate Viruses

Despite the large number of viruses discovered, we mainly focused on the characteristics of vertebrate viruses, which signified birds or mammals as virus hosts. In addition to these insect, plant, and fungal virus families (Luo et al., 2018; Wu et al., 2018), viruses of families *Rhabdoviridae*, *Phasmaviridae* and *Phenuiviridae* appeared to be insect viruses, because they were grouped with invertebrate RNA viruses in the phylogenetic trees (Supplementary Figure 4). Thus, we mainly describe the characteristics and phylogenetic relationships of viruses within families *Astroviridae*, *Hepeviridae*, *Nairoviridae*, *Picornaviridae* and *Picobirnaviridae*.

### Astroviridae

**FIGURE 2:**
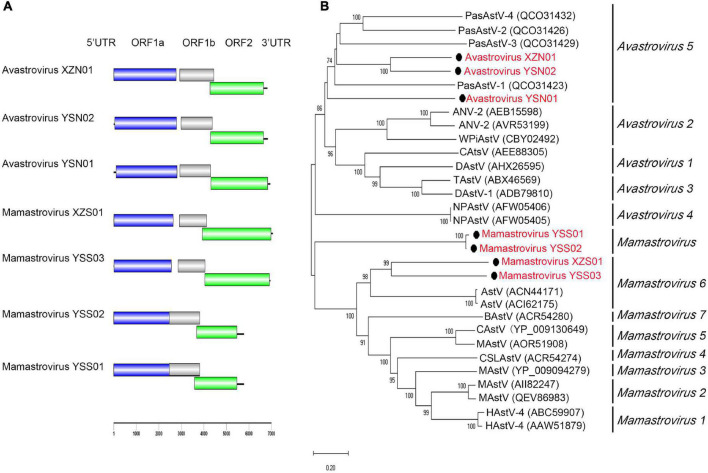
Genomic characterization and phylogenetic analysis of astroviruses. **(A)** Genomic characterization of astroviruses. **(B)** Phylogenetic analysis based on capsid amino acid sequences in the family *Astroviridae*. Phylogenetic tree was constructed using the maximum-likelihood method with 1,000 bootstrap replicates. The best-fit substitution model was Dayhoff. Only bootstrap values >70% are shown. Scale bar indicates nucleotide substitutions per site. Viruses in this study are indicated by solid black circles and in red font.

Seven nearly complete astrovirus genomes (length ranging from 5,779 to 7,066 bp) were detected and assembled from the four libraries. Avastrovirus YSN01 and YSN02 were identified in one bird (*Montifringilla taczanowskii*) fecal sample from Qinghai Province. Mamastrovirus YSS01–YSS03 were detected in one (*Apodemus peninsulae*), two (*A. peninsulae* and *Cricetulus kamensis*), and two (*A. peninsulae* and *Crocidura* sp.) fecal samples from Qinghai Province, respectively. Mamastrovirus XZS01 and avastrovirus XZN01 were detected from six rat fecal samples (all positive samples were of *Phaiomys leucurus*) and one bird (*Leucosticte brandti*) fecal sample, respectively (Supplementary Table 4). Each of these detected astroviruses showed typical genome organization comprising a single-stranded positive RNA containing three ORFs. ORF1a and ORF1b encoded non-structural polyproteins (a protease and an RdRp), and ORF2 encoded the viral capsid precursor (Figure 2A). According to the phylogenetic analysis (Figure 2B) based on ORF2 protein sequences, avastrovirus YSN01, YSN02, and XZN01 were assigned to species *Avastrovirus* 5 of the genus *Avastrovirus* (Fernández-Correa et al., 2019), whereas, mamastrovirus YSS01 and YSS02 formed an independent clade. The genetic distances (p-dist) and amino acid identity of ORF2 between mamastrovirus YSS01, YSS02, and their closely related members were greater than the threshold value 0.741 and less than 75%, respectively, indicating that mamastrovirus YSS01 and YSS02 may represent a new species of genus *Mamastrovirus*. Mamastrovirus YSS03 and XZS01 were closely related to members of species *Mamastrovirus* 6 (Alves et al., 2018), but their p-dist (>0.741) and amino acid identity (<75%) values shared with closely related members indicate that they represent new species belonging to genus *Mamastrovirus*.

### Hepeviridae

**FIGURE 3:**
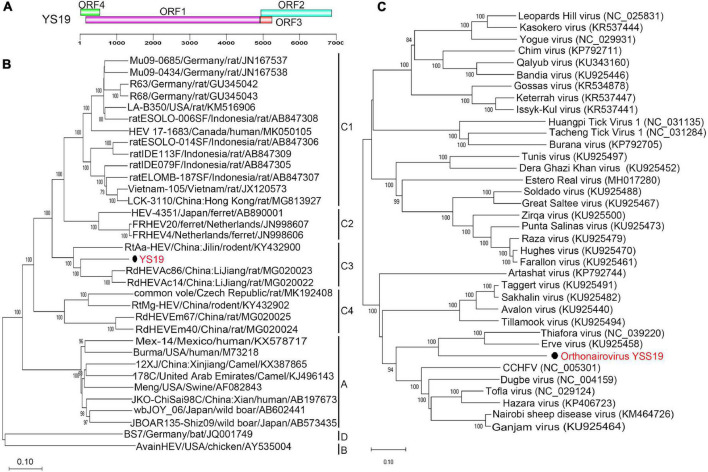
Genomic characterization and phylogenetic analysis of orthohepeviruses and orthonairoviruses. **(A)** Genomic characterization of *Orthohepevirus C* strain YS19. **(B)** Phylogenetic analysis using genomes of representatives within genus *Orthohepevirus*. Only bootstrap values >80% are shown. **(C)** Phylogenetic analysis based on amino acid sequences of the L segment from all species in genus *Orthonairovirus*. Bootstrap values (≥ 90%) are shown along branches. Each phylogenetic tree was constructed using the maximum-likelihood method with 1000 bootstrap replicates. The best-fit substitution models were JC and Dayhoff, respectively. Scale bar indicates nucleotide substitutions per site. Viruses in this study are indicated by solid black circles and in red font. CCHFV, *Crimean-Congo hemorrhagic fever orthonairovirus*.

In the YSSCD library, one highly divergent virus (7,934 bp; MW826539) of the family *Hepeviridae* was identified that showed only 37.2% amino acid identity to known *Hepeviridae* sp. muf159hep1. Another nearly complete genome (6,940 bp; MW391926) was obtained and named *Orthohepevirus C* strain YS19. The virus was detected in two fecal samples of *Ochotona curzoniae* and *A. peninsulae* from Qinghai Province. Complete genome analysis showed that *Orthohepevirus C* strain YS19 was closely related to *Orthohepevirus C* isolates RdHEVAc86 (77.6% nucleotide identity) and RdHEVAc14 (77.3% nucleotide identity). The genome organization of YS19 shows four ORFs similar to those of RdHEVAc86 and RdHEVAc14 (Figure 3A and Supplementary Figure 5). The amino acid identity between YS19 and RdHEVAc86 and RdHEVAc14 ranged from 70 to 91% (Supplementary Table 5). A genome-based maximum-likelihood phylogenetic tree revealed that *Orthohepevirus C* strain YS19 formed an independent cluster along with RdHEVAc86 and RdHEVAc14 within the group of the species *Orthohepevirus C* (Figure 3B). Values of pairwise distances between YS19 and RdHEVAc86 and RdHEVAc14 were 0.068 and 0.069, respectively, which were below the threshold of 0.088 (Smith et al., 2016). These results indicate that YS19 belongs to the HEV-C3, which includes RdHEVAc86 and RdHEVAc14.

### Nairoviridae

We identified an orthonairovirus in the family *Nairoviridae* from the YSSCD library, which was confirmed by PCR amplification using three paired primers (Supplementary Table 2). All three segments were found in only one shrew fecal sample from the Qinghai Province. The virus was named orthonairovirus YSS19 (YSV) under accession numbers MW391927–MW391929. The amino acid identity analysis indicated a close relationship of YSV with *Erve orthonairovirus* (ERVEV, 52.3–59.7%), *Thiafora orthonairovirus* (TFAV, 51.2–59.4%), and *Crimean-Congo hemorrhagic fever orthonairovirus* (CCHFV, 47.6–48.8%). Phylogenetic analysis based on the putative protein sequence of the L segment showed that YSV grouped with ERVEV and TFAV, which were closely related to CCHFV (Figure 3C). In addition, phylogenetic analysis based on putative protein sequences of M and S segments also showed similar results (Supplementary Figures 6, 7).

Codon usage preferences of YSV, ERVEV, TFAV, and CCHFV showed a highly similar pattern (Supplementary Figure 8). The glycoprotein precursor of YSV includes one unique proteolytic site and another identical to the proteolytic site of CCHFV and Nairobi sheep disease virus (NSDV) groups. The ovarian tumor domain (Frias-Staheli et al., 2007; Dilcher et al., 2012) of YSV (116 amino acids) shared 60.2%, 55.9%, and 45.8% amino acid identity with domains of ERVEV, TFAV, and CCHFV, respectively (Supplementary Figure 9).

### Picornaviridae

**FIGURE 4:**
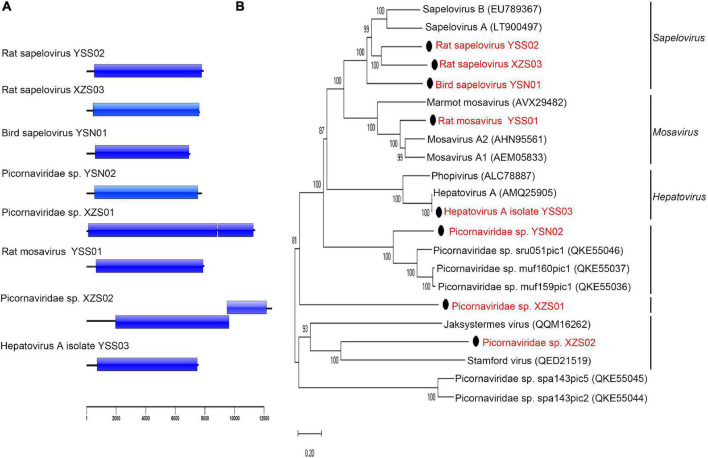
Genomic characterization and phylogenetic analysis of viruses belonging to *Picornaviridae*. **(A)** Genomic characterization of picorna-like viruses in this study. **(B)** Phylogenetic analysis based on amino acid sequences of the polyprotein of the family *Picornaviridae*. The phylogenetic tree was constructed using the maximum-likelihood method with 1000 bootstrap replicates. The best-fit substitution model was Dayhoff. Only bootstrap values >80% are shown. Scale bar indicates nucleotide substitutions per site. Viruses in this study are indicated by solid black circles and in red font.

Eight nearly complete picorna-like virus genomes were obtained (Figure 4A). Three viruses [*Picornaviridae* sp. XZS01 and XZS02, and rat sapelovirus (SPV) XZS03] were detected in rat fecal samples from Tibet Province, three viruses [rat mosavirus YSS01, rat SPV YSS02, and hepatovirus A (HAV) isolate YSS03] were identified in rat fecal samples from Qinghai Province, and two viruses (bird SPV YSN01 and *Picornaviridae* sp. YSN02) were found in bird fecal samples from Qinghai Province. A virus with <33% and 36% amino acid identity in P1 protein and 2C + 3 CD proteins may belong to a new genus of the family *Picornaviridae* (Zell et al., 2017). Rat SPV YSS02 and XZS03 shared 55.0% and 61.4% polyprotein amino acid identity with known members of genus *Sapelovirus*, indicating that they represent putative new species of that genus (Figure 4B). Polyprotein of rat mosavirus YSS01 shared 61.4% amino acid identity to that of *Mosavirus A2*. *Picornaviridae* sp. YSN02 shared 50.9% polyprotein amino acid identity with known *Picornaviridae* sp. sru051pic1. Bird SPV YSN01, *Picornaviridae* sp. XZS01, and *Picornaviridae* sp. XZS02 shared <40.5%, <38.7%, and <31.4% polyprotein amino acid identity with all known viruses of the family *Picornaviridae*, respectively, indicating that these three viruses may belong to putative new species or genera in family *Picornaviridae*.

HAV YSS03 (7,575 bp; MW391925) was identified in two samples from *A. peninsulae*; its genome included a 719-nucleotide 5′-untranslated region, 6756-nucleotide ORF-encoding polyprotein precursor (2,251 amino acids), and 100-nucleotide 3′-untranslated region. Sequence comparison of the HAV YSS03 genome showed 98.1% and 97.8% nucleotide similarities with Marmota himalayana hepatovirus (MHHAV) 2ID and 3ID, respectively. Interestingly, HAV YSS03 also shared 99.6% and 99.3% amino acid identity with MHHAV 2ID and 3ID, respectively. The potential polyprotein cleavage sites were identical between HAV YSS03 and MHHAV (Yu et al., 2016). Phylogenetic analysis based on genomic sequences (Supplementary Figure 10) revealed that HAV YSS03 clustered with MHHAV forming an independent branch within genus *Hepatovirus*. These results indicated that a nearly identical hepatovirus was present in sympatric yet genetically different hosts.

### Picobirnaviridae

**FIGURE 5:**
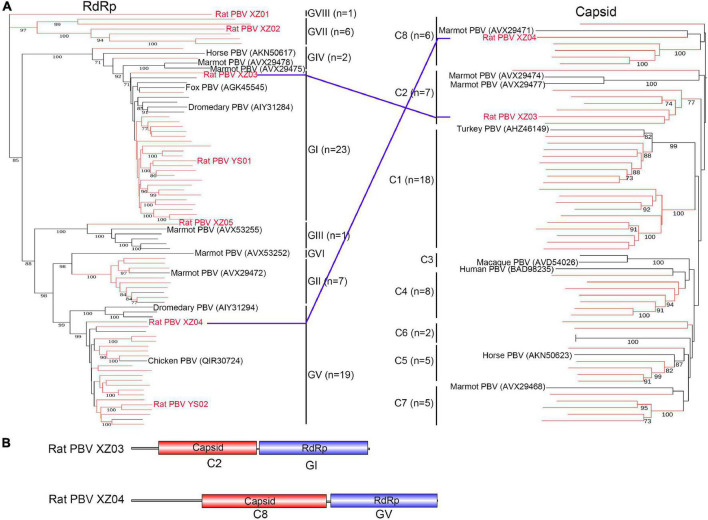
Phylogenetic analysis and genomic characterization of *Picobirnavirus*. **(A)** Phylogenetic analysis based on amino acid sequences of RNA-dependent RNA polymerase and capsid of genus *Picobirnavirus*. Each phylogenetic tree was constructed using the maximum-likelihood method with 1000 bootstrap replicates. The best-fit substitution model was Dayhoff. Only bootstrap values >70% are shown. Viruses in this study are indicated by red font and/or red branches. The two unsegmented picobirnaviruses are also indicated by blue lines. **(B)** Genomic characterization of the two unsegmented picobirnaviruses. PBV, *Picobirnavirus*.

The family *Picobirnaviridae* includes only one genus, *Picobirnavirus*, which is usually found in vertebrate fecal samples (Luo et al., 2018). Viruses possess bi-segmented double-stranded RNA genomes (i.e., segments 1 and 2) and are rarely unsegmented (Luo et al., 2018; Delmas et al., 2019). A total of 29 segment 1 (containing the RdRp domain) and 39 segment 2 (capsid) sequences were identified in rat fecal samples from Tibet Province with the addition of two unsegmented picobirnaviruses (PBVs). Moreover, a total of 28 segment 1 and nine segment 2 sequences were identified in rat fecal samples from Qinghai Province. The amino acid identity of RdRp with that of known PBVs was <83.6%, including only two RdRp sequences showing >80% identity. To investigate the evolutionary position of these PBVs, phylogenetic trees based on RdRp and capsid amino acid sequences were constructed (Figure 5A), and they revealed that newly discovered capsid sequences belong to eight clusters (C1–C8). In addition to known genotypes (GI–GVI), these newly discovered RdRp sequences also formed two new genogroups (GVII and GVIII). The six members of the GVII genogroup (Figure 5A) were detected in fecal samples from Tibet Province, sharing 23.6–43.1% amino acid identity with known PBVs. The GVIII genogroup contained only one virus (Rat PBV XZ01) with 48.1% amino acid identity with its closest relative. Interestingly, two unsegmented PBVs, named rat PBV XZ03 (3,494 bp) and rat PBV XZ04 (4,494 bp), were identified and had complete ORFs encoding the RdRp and capsid regions. The assortment types (Figure 5B) of rat PBV XZ03 (C2: GI) and rat PBV XZ04 (C8: GV) were different from those previously reported (Luo et al., 2018). Rat PBV XZ03 was detected in two rat (*Phaiomys leucurus*) fecal samples, and rat PBV XZ04 was detected in one fecal sample.

### Zoonotic Prediction

**FIGURE 6:**
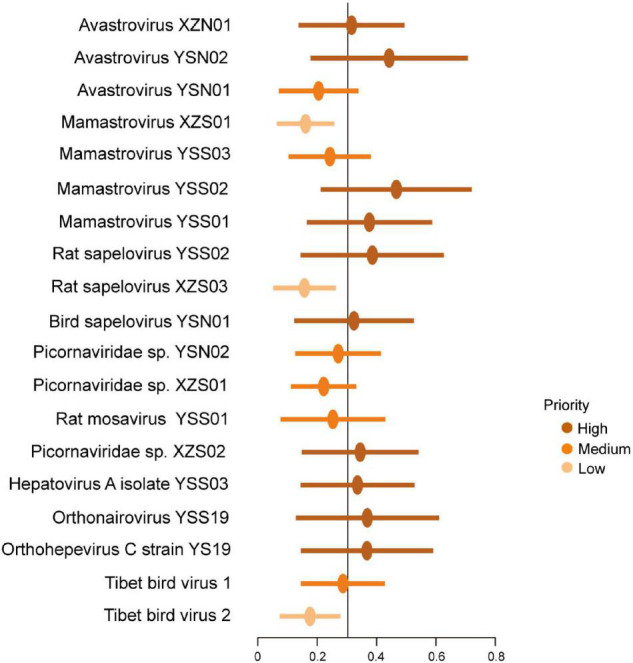
Probability of human infection based on viral genomes. Points reveal the mean calibrated score, with lines indicating 95% confidence intervals. The black line indicates a cutoff at 0.303.

Zoonotic risk (i.e., the probability of being able to infect humans) of newly discovered vertebrate viruses was evaluated as previously reported (Mollentze et al., 2021). Ten viruses (avastrovirus XZN01, avastrovirus YSN02, mamastrovirus YSS02, mamastrovirus YSS01, rat SPV YSS02, bird SPV YSN01, *Picornaviridae* sp. XZS02, HAV YSS03, YSV, and *Orthohepevirus C* strain YS19) were high priority (Figure 6). Mamastrovirus XZS01, rat SPV XZS03, and Tibet bird virus 2 were ranked as low priority (Figure 6).

### Invertebrate RNA Viruses

In addition to the viruses described above, a large number of invertebrate RNA viruses were detected. These viruses belonged to the families *Partitiviridae* (*n* = 23), *Solemoviridae* (*n* = 21), *Iflaviridae* (*n* = 8), *Dicistroviridae* (*n* = 7), *Nodaviridae* (*n* = 7) and *Totiviridae* (*n* = 6) (Figure 1). Also, 12 of these viruses were distantly related to previously known viruses and each other. The RdRp domain of these viruses shared 26.3–37.6% amino acid identity with corresponding sequences of their closest relatives (Table 1), indicating that these 12 viruses may belong to potentially new genera or families.

**TABLE 1:** Contig length, amino acid identity, and closest relative of the divergent viruses detected in this study.

Virus name	Accession numbers	Length of RdRp or polyprotein (amino acids)	Samples (pool)	Amino acid identity (%) [closest relative]
*Arlivirus* sp. YSN1024	MW826497	2160	bird (YSNCD)	32.2 [Hemipteran arli-related virus OKIAV95]
*Arlivirus* sp. XZN142933	MW864073	1195	bird (XZNCD)	34.1 [Lishi spider virus 2]
*Comovirus* sp. 143027	MW930299	1829	bird (XZNCD)	31.6 [Phaseolus vulgaris severe mosaic virus]
*Dicistroviridae* sp. XZN128099	MW826400	1800	bird (XZNCD)	35.7 [*Dicistroviridae* sp.]
*Iflaviridae* sp. XZN178790	MW826394	2998	bird (XZNCD)	29.6 [Vespa velutina associated ifla-like virus]
*Levivirida*e sp. XZS180134	MW826429	615	rat (XZSCD)	33.8 [*Leviviridae* sp.]
*Mitovirus* sp. XZS182170	MW826428	830	rat (XZSCD)	27.8 [Plasmopara viticola lesion associated mitovirus 39]
*Mitovirus* sp. XZS182324	MW826426	872	rat (XZSCD)	29.2 [Gergich narna-like virus]
*Nodaviridae* sp. XZS178253	MW826427	504	rat (XZSCD)	37.0 [Nodaviridae sp.]
*Nodaviridae* sp. YSN11758	MW826486	524	rat (YSNCD)	37.6 [*Nodaviridae* sp.]
*Polycipiviridae* sp. XZN137140	MW826414	2559	bird (XZNCD)	30.0 [Nuksystermes virus]
*Polycipiviridae* sp. XZN136291	MW826415	2813	bird (XZNCD)	26.3 [Yongsan picorna-like virus 3]

## Discussion

The SARS-CoV-2 pandemic highlighted the need to investigate previously unknown viruses from wild animals in an unbiased manner (Albery et al., 2021; Grange et al., 2021; Hu et al., 2021). Information on viral diversity in animals, especially in birds and small mammals from the Qinghai–Tibet Plateau (average altitude >4000 m), is still limited. Here, we identified 184 RNA viruses with low prevalence rate from birds and small mammals, which belong to 28 virus families, suggesting a higher viral diversity in the Qinghai–Tibet Plateau.

These new viruses extend our understanding of RNA virus diversity in wild animals from the highest and largest plateau in the world. We could identify new genera of *Picornaviridae*, new species of genera *Mamastrovirus* and *Orthonairovirus*, and new genogroups of *Picobirnavirus* in these samples. We also discovered a new variant (orthohepevirus strain YS19) belonging to the HEV-C3 genotype that was clustered with RdHEVAc86, RdHEVAc14, and RtAa-HEV/JL2014. In addition, invertebrate RNA viruses that were significantly distant from known genera or families increase the number of viral taxa, providing a basis for further virus identification.

This study also broadens our knowledge in hosts of known RNA virus. First, we identified a hepatovirus (hepatovirus A isolate YSS03) that was identical to previously reported MHHAV (99.3–99.6% amino acid identity) causing fever in *M. himalayana* (Yu et al., 2016). The nearly identical hepatovirus was detected in the samples from the same region but genetically different hosts (Yu et al., 2016), suggesting a possible cross-host transmission and circulation between *M. himalayana* and *A. peninsulae* in the region. Second, a virus of genus *Tobamovirus* in the family *Virgaviridae* (accession number MW826405) was identified in a bird fecal sample from Tibet Province. It shared 100% RdRp amino acid identity with pepper mild mottle virus (accession number QIM41079) from *Nicotiana occidentalis* in Slovenia. Third, a large number of new PBVs were identified in rat fecal samples from Tibet and Qinghai Provinces, which indicates that the rat is an important wildlife host for PBV.

Although zoonotic potential is estimated based only on genomic signatures, genome-based ranking may shed new light on further studies. The training datasets contained genome sequences of 861 virus species within 36 families, which were known to infect humans as previously reported (Olival et al., 2017; Woolhouse and Brierley, 2018; Mollentze et al., 2021). The predicted probability was trained and calculated on genomic signatures (146 measures), such as dinucleotide biases, amino acid biases and relative frequency of each codon (Mollentze et al., 2021). The cut-off value (0.303) is the optimal balance between sensitivity and specificity in the dataset (Bergner et al., 2021; Mollentze et al., 2021). The best model is built based on generalizable signatures of virus genomes and combined information across viral families, and evaluated using genomes of 758 other virus species, which predicts more accurately than those based on relatedness (e.g., taxonomy) based models (Ladner, 2021). In addition, although compositional similarity may influence predictions, no individual or specific characteristic of viral genomes is primarily responsible for ranking zoonotic potential, with complex and non-linear relationships among characteristics (Mollentze et al., 2021). Thus, the genome-based ranking could be stable and reliable. However, we should acknowledge that the zoonotic potentials of these viruses are still preliminary and need additional confirmatory testing.

## Conclusion

In conclusion, the RNA viromes of birds and small mammals were characterized, providing a fresh perspective on the viral diversity in the Qinghai–Tibet Plateau. Because of the global diversity and distribution of rodent and bird viruses, it is crucial to pay more attention to their role in viral diseases.

## Data Availability Statement

All genome sequences were submitted to GenBank under accession numbers MW391925-MW391929, MW826371-MW 826560, MW864073-MW864077, and MW930234-MW930300, respectively.

## Ethics Statement

The animal study was reviewed and approved by the Ethics Committee of National Institute for Communicable Disease Control and Prevention, China CDC.

## Author Contributions

JX, WZ, and ZL contributed to conception and designed of the study. WZ, SL, JY, and SW collected the samples. WZ performed the experiments. WZ, JP, DJ, and X-LL performed the statistical analysis. WZ and JY wrote the first draft of the manuscript. JX, JY, and LL acquired the funding. All authors contributed to the article and approved the submitted version.

## Conflict of Interest

The authors declare that the research was conducted in the absence of any commercial or financial relationships that could be construed as a potential conflict of interest.

## Publisher’s Note

All claims expressed in this article are solely those of the authors and do not necessarily represent those of their affiliated organizations, or those of the publisher, the editors and the reviewers. Any product that may be evaluated in this article, or claim that may be made by its manufacturer, is not guaranteed or endorsed by the publisher.

## Footnotes

1
https://ftp.ncbi.nlm.nih.gov/genomes/Viruses/

2
https://www.ncbi.nlm.nih.gov/orffinder/

3
https://www.ncbi.nlm.nih.gov/cdd

4
https://cousin.ird.fr/
