# Supplementary material for: RNA Virus Diversity in Birds and Small Mammals From Qinghai–Tibet Plateau of China
Source: Front Microbiol. 2022 Feb 18;13:780651. doi: 10.3389/fmicb.2022.780651 (PMC8894885; doi:10.3389/fmicb.2022.780651)
Supplement: Supplementary file 1 [file Data_Sheet_1.docx]

**RNA virus diversity in birds and small mammals from**

**Qinghai-Tibet plateau of China**

Wentao Zhu^1^, Jing Yang^1,2,3^, Shan Lu^1,2,3^, Dong Jin^1,2,3^, Ji Pu^1^, Shusheng Wu^4^, Xue-lian Luo^1^, Liyun Liu^1^, Zhenjun Li^1^ and Jianguo Xu^1,2,3,5*^

1. State Key Laboratory of Infectious Disease Prevention and Control, National Institute for Communicable Disease Control and Prevention, Chinese Center for Disease Control and Prevention, Changping, Beijing 102206, PR China;

2. Shanghai Public Health Clinical Center, Fudan University, Shanghai 201508, PR China;

3. Research Units of Discovery of Unknown Bacteria and Function, Chinese Academy of Medical Sciences, Beijing 100730, PR China;

4. Yushu Prefecture Center for Disease Control and Prevention, China

5. Research Institute of Public Heath, Nankai University, Tianjin 300071, China

*Correspondence author: Jianguo Xu. Email: xujianguo@icdc.cn

Table S1 The animal species, sampling location and dates used in this study

| **Animal class (numbers)** | **Animal species** | **Date** | **Location** |
| --- | --- | --- | --- |
| Birds (31) | *Leucosticte brandti* | 2018.4 | Cona County, Tibet Province |
| Rats (41) | *Phaiomys leucurus* | 2018.4 | Cona County, Tibet Province |
| Birds (25) | *Montifringilla taczanowskii*, *Pseudopodoces humilis* | 2019.7 | Yushu County, Qinghai Province |
| Small mammals (50) | *Apodemus peninsulae*, *Ochotona curzoniae*, *Lasiopodomys fuscus*, *Cricetulus kamensis*, *Crocidura* sp. | 2019.7 | Nangqian County, Qinghai Province |

Table S2 Primers used in this study

| **Viruses** | **Primers** | **Sequences** | **Length (bp)** | **Tm (℃)** |
| --- | --- | --- | --- | --- |
| Tibet bird virus 1 | Phas-F | 5’-CGGTTGCTAAAACACTGGCA-3’ | 725 | 60 |
|  | Phas-R | 5’-TCGCCTAGCAGACTGGGTAT-3’ |  |  |
| Tibet bird virus 2 | Phen-F | 5’-CTTCACATGGTGAGCCTGGT-3’ | 539 | 60 |
|  | Phen-R | 5’-GACTGTCCCGATGGACTTGG-3’ |  |  |
| Rat Picobirnavirus XZ03 | Picobir-F1 | 5’-GTGCACCCAATCCTAACCCA-3’ | 569 | 60 |
|  | Picobir-R1 | 5’-TTTGGGCACAGCTAGGGATG-3’ |  |  |
| Rat Picobirnavirus XZ04 | Picobir-F2 | 5’-TCAGAAGATTGCCAGCGTGT-3’ | 782 | 60 |
|  | Picobir-R2 | 5’-GATAAAGCTGCTGCGTCGTG-3’ |  |  |
| Avastrovirus XZN01 | Astro-F1 | 5’-TGTGCACTGACCCAACCTTT-3’ | 768 | 60 |
|  | Astro-R1 | 5’-CTTCCAGAGAGGGTAGGGCT-3’ |  |  |
| Mamastrovirus XZS01 | Astro-F2 | 5’-TCCACAACCTGCACAACCTT-3’ | 767 | 60 |
|  | Astro-R2 | 5’-AAGTGAGACACGGACAGCAG-3’ |  |  |
| Avastrovirus YSN02 | Astro-F3 | 5’-GCTGGAGAATTCGGGGTCAA-3’ | 742 | 60 |
|  | Astro-R3 | 5’-AGTCGGCCGAAGCCAATTTA-3’ |  |  |
| Avastrovirus YSN01 | Astro-F4 | 5’-CAGACCACGCACACTGGTAT-3’ | 719 | 60 |
|  | Astro-R4 | 5’-AAGGACCTGCGGATCTTTGG-3’ |  |  |
| Mamastrovirus YSS03 | Astro-F5 | 5’-GCACGAACCACCAAAGGAAC-3’ | 637 | 60 |
|  | Astro-R5 | 5’-TTGGGTCGGCACAGAGAATC-3’ |  |  |
| Mamastrovirus YSS01 | Astro-F6 | 5’-GCACCTCTGACTTCGAGCTT-3’ | 615 | 60 |
|  | Astro-R6 | 5’-AGCCGAGGTAGTCTGTGAGT-3’ |  |  |
| Mamastrovirus YSS02 | Astro-F7 | 5’-CTACCGTCATGCTCCATCCC-3’ | 785 | 60 |
|  | Astro-R7 | 5’-CTTAGCGGCGTCAAAATCGG-3’ |  |  |
| Hepatovirus A | Hepa-F | 5’-GATCCACAATATCCAGTTTGGG-3’ | 805 | 55 |
|  | Hepa-R | 5’-CATGGTGTGCCACATTACTAGG-3’ |  |  |
| Orthohepevirus C | HEV-F | 5’-GTCAACCACCGTCAAACGTG-3’ | 939 | 55 |
|  | HEV-R | 5’-CAATGCACGGCATCGGTAAG-3’ |  |  |
| Orthonairovirus  L segment | NaiL-F | 5’-AAGAGATGGATGACTTTGAA-3’ | 1842 | 49 |
|  | NaiL-R | 5’-TGACCATCTCATAAACAACTT-3’ |  |  |
| Orthonairovirus  M segment | NaiM-F | 5’-ACAACACTTGGATGTCCTGGC-3’ | 1036 | 59 |
|  | NaiM-R | 5’-AGGATTGATGGCTGGTGTGG-3’ |  |  |
| Orthonairovirus  S segment | NaiS-F | 5’-CACCTTGTTTTGAGCGATGG-3’ | 2209 | 59 |
|  | NaiS-R | 5’-TCAAAGCTTATGGCACGCAC-3’ |  |  |
| Picornaviridae sp. XZS01 | Picorna-F1 | 5’-TGGCCTAATGAACGCACCAT-3’ | 977 | 60 |
|  | Picorna-R1 | 5’-TATGGGGGTTTTGCTCGCTT-3’ |  |  |
| Picornaviridae sp. XZS02 | Picorna-F2 | 5’-GGCCTCCATCTCCATTACCG-3’ | 765 | 60 |
|  | Picorna-R2 | 5’-ATGGAGCTGTTACCAAGGGC-3’ |  |  |
| Rat mosavirus YSS01 | Picorna-F3 | 5’-ACTGGAGGCAATTCGGTGAG-3’ | 729 | 60 |
|  | Picorna-R3 | 5’-CCTCGGTCGCCTCAGTAATC-3’ |  |  |
| Rat sapelovirus YSS02 | Picorna-F4 | 5’-TGGTTTGAGACAGAGCGTCC-3’ | 792 | 60 |
|  | Picorna-R4 | 5’-GCGTTTTGCACTTCGGTCTT-3’ |  |  |
| Rat sapelovirus XZS03 | Picorna-F5 | 5’-GGCCTGTCTATTTGGGCACT-3’ | 999 | 60 |
|  | Picorna-R5 | 5’-CTGTAATGGCATTTCGGGCG-3’ |  |  |
| Picornaviridae sp. YSN02 | Picorna-F6 | 5’-GGCCTGTCTATTTGGGCACT-3’ | 999 | 60 |
|  | Picorna-R6 | 5’-CTGTAATGGCATTTCGGGCG-3’ |  |  |
| Bird sapelovirus YSN01 | Picorna-F7 | 5’-TGTAACGCCCCAAGCTGATT-3’ | 745 | 60 |
|  | Picorna-R7 | 5’-ATTCCCTTGCGTCTCCACAG-3’ |  |  |
| **The following primers are used to fill gaps** | | | | |
| Hepatovirus A | BuF1 | GATAAGAATGAAACTGTTGAGAAA | 993 | 53 |
|  | BuR1 | AAGATAACACACTGTAGGCTCAC |  |  |
| Hepatovirus A | BuF2 | TGTAGAATGTTTTGGGGTCC | 886 | 55 |
|  | BuR2 | TAATCAAAATCTCTAAAAAAATCCA |  |  |
| Rat Picobirnavirus XZ04 | Picobir F21 | CACGACGCAGCAGCTTTATC | 315 | 60 |
|  | Picobir R21 | AGCGAGCCGTTTTGCAATTT |  |  |

The amplifications were conducted under the following conditions: 94°C for 5 min, followed by 30 cycles (94°C for 30 s, Tm–5°C for 30 s, and 72°C for 1 min/kb) and then 72°C for 7 min.

Table S3 Viruses identified in this study with their corresponding accession numbers

| **Virus family** | **Virus name** | **Accession**  **number** | **Virus family** | **Virus name** | **Accession number** |
| --- | --- | --- | --- | --- | --- |
| *Astroviridae* | Avastrovirus XZN01 | MW826371 | *Picobirnaviridae*  *Picobirnaviridae*  *Picobirnaviridae* | Rat Picobirnavirus YS12 RdRp | MW930251 |
|  | Mamastrovirus XZS01 | MW826416 |  | Rat Picobirnavirus YS13 RdRp | MW930252 |
|  | Avastrovirus YSN02 | MW826482 |  | Rat Picobirnavirus YS14 RdRp | MW930253 |
|  | Avastrovirus YSN01 | MW826483 |  | Rat Picobirnavirus YS15 RdRp | MW930254 |
|  | Mamastrovirus YSS03 | MW826530 |  | Rat Picobirnavirus YS16 RdRp | MW930255 |
|  | Mamastrovirus YSS01 | MW826531 |  | Rat Picobirnavirus YS17 RdRp | MW930256 |
|  | Mamastrovirus YSS02 | MW826532 |  | Rat Picobirnavirus YS18 RdRp | MW930257 |
| *Barnaviridae* | Barnaviridae sp. XZS182276 | MW826417 |  | Rat Picobirnavirus YS19 RdRp | MW930258 |
| *Bromoviridae* | Bromoviridae sp. YSN01 RNA3 | MW826484 |  | Rat Picobirnavirus YS20 RdRp | MW930259 |
|  | Bromoviridae sp. YSN01 RNA2 | MW826485 |  | Rat Picobirnavirus YS21 RdRp | MW930260 |
|  | Bromoviridae sp. YSN01 RNA1 | MW826506 |  | Rat Picobirnavirus YS22 RdRp | MW930261 |
| *Chuviridae* | Chuviridae sp. XZN143138 RNA1 | MW826401 |  | Rat Picobirnavirus YS23 RdRp | MW930262 |
|  | Chuviridae sp. XZN143138 RNA2 | MW826402 |  | Rat Picobirnavirus YS24 RdRp | MW930263 |
|  | Chuviridae sp. XZS178169 | MW826430 |  | Rat Picobirnavirus YS25 RdRp | MW930264 |
| *Dicistroviridae* | Dicistroviridae sp. XZN142635 | MW826398 |  | Rat Picobirnavirus YS26 RdRp | MW930265 |
|  |  |  |  | Rat Picobirnavirus YS27 RdRp | MW930266 |
|  | Dicistroviridae sp. XZN128099 | MW826400 |  | Rat Picobirnavirus YS28 RdRp | MW930267 |
|  | Dicistroviridae sp. YSN714 | MW826502 |  | Rat Picobirnavirus XZS153052 | MW826431 |
|  | Dicistroviridae sp. YSN11649 | MW826503 |  | Rat Picobirnavirus XZS162693 | MW826432 |
|  | Dicistroviridae sp. YSN401 | MW826504 |  | Rat Picobirnavirus XZS171157 | MW826433 |
|  | Dicistroviridae sp. YSN16443 | MW826505 |  | Rat Picobirnavirus XZS172287 | MW826434 |
|  | Dicistroviridae sp. YSS10464 | MW826540 |  | Rat Picobirnavirus XZS172337 | MW826435 |
| *Hepeviridae* | Orthohepevirus C strain YS19 | MW391926 |  | Rat Picobirnavirus XZS173032 | MW826436 |
|  | Hepeviridae sp. YSS66421 | MW826539 |  | Rat Picobirnavirus XZS174378 | MW826437 |
| *Iflaviridae* | Iflaviridae sp. XZN142898 | MW826391 |  | Rat Picobirnavirus XZS175385 | MW826438 |
|  | Iflaviridae sp. XZN142719 | MW826392 |  | Rat Picobirnavirus XZS175479 | MW826439 |
|  | Iflaviridae sp. XZN143281 | MW826393 |  | Rat Picobirnavirus XZS177003 | MW826440 |
|  | Iflaviridae sp. XZN139912 | MW864074 |  | Rat Picobirnavirus XZS177362 | MW826441 |
|  | Iflaviridae sp. XZN178790 | MW826394 |  | Rat Picobirnavirus XZS177391 | MW826442 |
|  | Iflaviridae sp. XZN321 | MW826395 |  | Rat Picobirnavirus XZS177758 | MW826443 |
|  | Iflaviridae sp. XZN440 | MW826396 |  | Rat Picobirnavirus XZS177905 | MW826444 |
|  | Iflaviridae sp. XZN9751 | MW826397 |  | Rat Picobirnavirus XZS178320 | MW826445 |
| *Leviviridae* | Leviviridae sp. XZS180134 | MW826429 |  | Rat Picobirnavirus XZS178780 | MW826446 |
| *Lispiviridae* | Arlivirus sp. YSN1024 | MW826497 |  | Rat Picobirnavirus XZS179077 | MW826447 |
|  | Arlivirus sp. XZN142933 | MW864073 |  | Rat Picobirnavirus XZS179369 | MW826448 |
| *Mitoviridae* | Mitovirus sp. XZS182170 | MW826428 |  | Rat Picobirnavirus XZS179412 | MW826449 |
|  | Mitovirus sp. XZS182324 | MW826426 |  |  |  |
| *Nairoviridae* | Orthonairovirus L segment | MW391927 |  | Rat Picobirnavirus XZS179415 | MW826450 |
|  | Orthonairovirus M segment | MW391928 |  | Rat Picobirnavirus XZS179448 | MW826451 |
|  | Orthonairovirus S segment | MW391929 |  | Rat Picobirnavirus XZS179569 | MW826452 |
| *Narnaviridae* | Narnaviridae sp. XZS181226 | MW826425 |  | Rat Picobirnavirus XZS179781 | MW826453 |
| *Nodaviridae* | Nodaviridae sp. XZN35167 RNA1 | MW826389 |  | Rat Picobirnavirus XZS179869 | MW826454 |
|  | Nodaviridae sp. XZN35167 RNA2 | MW826390 |  | Rat Picobirnavirus XZS179900 | MW826455 |
|  | Nodaviridae sp. XZS178253 | MW826427 |  | Rat Picobirnavirus XZS180083 | MW826456 |
|  | *Nodaviridae* sp. YSN11758 | MW826486 |  | Rat Picobirnavirus XZS180089 | MW826457 |
|  | Nodaviridae sp. YSN261 RNA1 | MW826491 |  | Rat Picobirnavirus XZS180128 | MW826458 |
|  | Nodaviridae sp. YSN261 RNA2 | MW826492 |  | Rat Picobirnavirus XZS180155 | MW826459 |
|  | Nodaviridae sp. YSN246 RNA1 | MW826493 |  | Rat Picobirnavirus XZS180285 | MW826460 |
|  | Nodaviridae sp. YSN246 RNA2 | MW826494 |  | Rat Picobirnavirus XZS180422 | MW826461 |
|  | Nodaviridae sp. YSN665 RNA1 | MW826495 |  | Rat Picobirnavirus XZS180918 | MW826462 |
|  | Nodaviridae sp. YSN665 RNA2 | MW826496 |  | Rat Picobirnavirus XZS181191 | MW826463 |
| *Partitiviridae* | Partitiviridae sp. XZN134567 | MW826372 |  | Rat Picobirnavirus XZS181207 | MW826464 |
|  | Partitiviridae sp. XZN134567 | MW826373 |  | Rat Picobirnavirus XZS182294 | MW826465 |
|  | Partitiviridae sp. XZN137934 | MW826374 |  | Rat Picobirnavirus XZS186777 | MW826466 |
|  | Partitiviridae sp. XZN137934 | MW826375 |  | Rat Picobirnavirus XZS209090 | MW826467 |
|  | Partitiviridae sp. XZS177413 | MW826418 |  | Rat Picobirnavirus XZS295413 | MW826468 |
|  | Partitiviridae sp. XZS177413 | MW826419 |  | Rat Picobirnavirus XZS65514 | MW826469 |
|  | Partitiviridae sp. XZS155894 | MW826420 |  | Rat Picobirnavirus YSS5088 | MW826541 |
|  | Partitiviridae sp. XZS155894 | MW826421 |  | Rat Picobirnavirus YSS2632 | MW826542 |
|  | Partitiviridae sp. XZS160380 | MW826422 |  | Rat Picobirnavirus YSS8037 | MW826543 |
|  | Partitiviridae sp. XZS160380 | MW826423 |  | Rat Picobirnavirus YSS4921 | MW826544 |
|  | Partitiviridae sp. XZN134888 | MW826376 |  | Rat Picobirnavirus YSS2597 | MW826545 |
|  | Partitiviridae sp. XZN137388 | MW826377 |  | Rat Picobirnavirus YSS5028 | MW826546 |
|  | Partitiviridae sp. XZN134375 | MW826378 |  | Rat Picobirnavirus YSS5576 | MW826547 |
|  | Partitiviridae sp. XZN135748 | MW826379 |  | Rat Picobirnavirus YSS135602 | MW826548 |
|  | Partitiviridae sp. YSN381 | MW826487 |  | Rat Picobirnavirus YSS4934 | MW826549 |
|  | Partitiviridae sp. YSN217 | MW826488 | *Picornaviridae* | Picornaviridae sp. XZS01 | MW826470 |
|  | Partitiviridae sp. YSN103 | MW826489 |  | Picornaviridae sp. XZS02 | MW826471 |
|  | Partitiviridae sp. YSN504 | MW826489 |  | Rat mosavirus YSS01 | MW826550 |
|  | Partitiviridae sp. YSS3194 | MW826533 |  | Rat sapelovirus YSS02 | MW826551 |
|  | Partitiviridae sp. YSS7261 | MW826534 |  | Rat sapelovirus XZS03 | MW826472 |
|  | Partitiviridae sp. YSS68631 | MW826534 |  | Picornaviridae sp. YSN02 | MW826507 |
|  | Partitiviridae sp. YSS9817 | MW826536 |  | Bird sapelovirus YSN01 | MW826529 |
|  | Partitiviridae sp. YSS5908 | MW826537 |  | Hepatovirus A isolate YSS03 | MW391925 |
|  | Partitiviridae sp. XZN138740 | MW826380 | *Polycipiviridae* | Polycipiviridae sp. XZN137140 | MW826414 |
|  | Partitiviridae sp. XZN141070 | MW826381 |  | Polycipiviridae sp. XZN141292 | MW864077 |
|  | Partitiviridae sp. XZN141309 | MW826382 |  | Polycipiviridae sp. YSN480 | MW826527 |
|  | Partitiviridae sp. XZN62437 | MW826383 |  | Polycipiviridae sp. YSN200 | MW826528 |
|  | Partitiviridae sp. XZN113441 | MW826384 |  | Polycipiviridae sp. XZN136291 | MW826415 |
|  | Partitiviridae sp. XZN117960 | MW826385 | *Reoviridae* | Reoviridae sp. YSN13 | MW826526 |
|  | Partitiviridae sp. XZN130727 | MW826386 |  | Reoviridae sp. XZN127658 | MW826413 |
|  |  |  | *Rhabdoviridae* | Rhabdoviridae sp. XZS177686 | MW826481 |
|  | Partitiviridae sp. YSS126390 | MW826538 |  | Rhabdoviridae sp. YSN900 | MW826525 |
|  | Partitiviridae sp. XZN137982 | MW826387 | *Secoviridae* | Comovirus sp. 143027 RNA1 | MW930299 |
|  | Partitiviridae sp. XZN138430 | MW826388 |  | Comovirus sp. 143027 RNA2 | MW930300 |
| *Phasmaviridae* | Tibet bird virus 1 L segment | MW930237 |  | Nepovirus sp. XZN143379 | MW826412 |
|  | Tibet bird virus 1 M segment | MW930238 |  | Nepovirus sp. XZS180819 | MW826478 |
|  | Tibet bird virus 1 S segment | MW930239 |  | Nepovirus sp. XZS182521 | MW826479 |
| *Phenuiviridae* | Tibet bird virus 2 L segment | MW930234 |  | Nepovirus sp. XZS18252 | MW826480 |
|  | Tibet bird virus 2 M segment | MW930235 | *Solemoviridae* | Solemoviridae sp. XZN127634 | MW826408 |
|  | Tibet bird virus 2 S segment | MW930236 |  | Solemoviridae sp. XZN143026 | MW826409 |
| *Picobirnaviridae*  *Picobirnaviridae* | Rat Picobirnavirus XZ03 RdRp | MW930294 |  | Solemoviridae sp. XZN79705 | MW826410 |
|  | Rat Picobirnavirus XZ04 RdRp | MW930295 |  | Solemoviridae sp. XZS181522 | MW826477 |
|  | Rat Picobirnavirus XZ01 RdRp | MW930296 |  | Solemoviridae sp. YSN14675 | MW826513 |
|  | Rat Picobirnavirus XZ02 RdRp | MW930297 |  | Solemoviridae sp. YSN909 | MW826514 |
|  | Rat Picobirnavirus XZ05 RdRp | MW930298 |  | Solemoviridae sp. YSN6450 | MW826515 |
|  | Rat Picobirnavirus XZ06 RdRp | MW930268 |  | Solemoviridae sp. YSN1673 | MW826516 |
|  | Rat Picobirnavirus XZ07 RdRp | MW930269 |  | Solemoviridae sp. YSN255 | MW826517 |
|  | Rat Picobirnavirus XZ08 RdRp | MW930270 |  | Solemoviridae sp. YSN551 | MW826518 |
|  | Rat Picobirnavirus XZ09 RdRp | MW930271 |  | Solemoviridae sp. YSN13713 | MW826519 |
|  | Rat Picobirnavirus XZ10 RdRp | MW930272 |  | Solemoviridae sp. YSN675 | MW826520 |
|  | Rat Picobirnavirus XZ11 RdRp | MW930273 |  | Solemoviridae sp. YSN7936 | MW826521 |
|  | Rat Picobirnavirus XZ12 RdRp | MW930274 |  | Solemoviridae sp. YSN1671 | MW826522 |
|  | Rat Picobirnavirus XZ13 RdRp | MW930275 |  | Solemoviridae sp. YSS62163 | MW826558 |
|  | Rat Picobirnavirus XZ14 RdRp | MW930276 |  | Solemoviridae sp. YSS54631 | MW826559 |
|  | Rat Picobirnavirus XZ15 RdRp | MW930277 |  | Solemoviridae sp. YSS54632 | MW826560 |
|  | Rat Picobirnavirus XZ16 RdRp | MW930278 |  | Solemoviridae sp. XZN132066 | MW826411 |
|  | Rat Picobirnavirus XZ17 RdRp | MW930279 |  | Solemoviridae sp. YSN169 | MW826523 |
|  | Rat Picobirnavirus XZ18 RdRp | MW930280 |  | Solemoviridae sp. YSN359 | MW826498 |
|  | Rat Picobirnavirus XZ19 RdRp | MW930281 |  | Solemoviridae sp. YSN6030 | MW826499 |
|  | Rat Picobirnavirus XZ20 RdRp | MW930282 |  | Solemoviridae sp. YSN933 | MW826500 |
|  | Rat Picobirnavirus XZ21 RdRp | MW930283 |  | Solemoviridae sp. YSN10169 | MW826501 |
|  | Rat Picobirnavirus XZ22 RdRp | MW930284 |  | Solemoviridae sp. YSN9007 | MW826524 |
|  | Rat Picobirnavirus XZ23 RdRp | MW930285 | *Tombusviridae* | Tombusviridae sp. YSN218 | MW826511 |
|  | Rat Picobirnavirus XZ24 RdRp | MW930286 |  | Tombusviridae sp. YSN286 | MW826512 |
|  | Rat Picobirnavirus XZ25 RdRp | MW930287 |  | Tombusviridae sp. YSS2851 | MW826553 |
|  | Rat Picobirnavirus XZ26 RdRp | MW930288 |  | Tombusviridae sp. YSS1786 | MW826554 |
|  | Rat Picobirnavirus XZ27 RdRp | MW930289 |  | Tombusviridae sp. YSS133061 | MW826555 |
|  | Rat Picobirnavirus XZ28 RdRp | MW930290 | *Totiviridae* | Totiviridae sp. YSN771 | MW826508 |
|  | Rat Picobirnavirus XZ29 RdRp | MW930291 |  | Totiviridae sp. XZN139620 | MW826406 |
|  | Rat Picobirnavirus XZ30 RdRp | MW930292 |  | Totiviridae sp. XZN140231 | MW826407 |
|  | Rat Picobirnavirus XZ31 RdRp | MW930293 |  | Totiviridae sp. XZS182394 | MW826476 |
|  | Rat Picobirnavirus YS01 RdRp | MW930240 |  | Totiviridae sp. YSN397 | MW826509 |
|  | Rat Picobirnavirus YS02 RdRp | MW930241 |  | Totiviridae sp. YSN140 | MW826510 |
|  | Rat Picobirnavirus YS03 RdRp | MW930242 | *Tymoviridae* | Tymoviridae sp. YSS133186 | MW826552 |
|  | Rat Picobirnavirus YS04 RdRp | MW930243 |  | Tymoviridae sp. XZN137671 | MW864076 |
|  | Rat Picobirnavirus YS05 RdRp | MW930244 | *Virgaviridae* | Virgaviridae sp. XZN126168 | MW864075 |
|  | Rat Picobirnavirus YS06 RdRp | MW930245 |  | Virgaviridae sp. XZN126168 | MW826403 |
|  | Rat Picobirnavirus YS07 RdRp | MW930246 |  | Virgaviridae sp. XZN126168 | MW826404 |
|  | Rat Picobirnavirus YS08 RdRp | MW930247 |  | Virgaviridae sp. XZS160203 | MW826473 |
|  | Rat Picobirnavirus YS09 RdRp | MW930248 |  | Virgaviridae sp. XZS160203 | MW826474 |
|  | Rat Picobirnavirus YS10 RdRp | MW930249 |  | Virgaviridae sp. XZS160203 | MW826475 |
|  | Rat Picobirnavirus YS11 RdRp | MW930250 |  | Virgaviridae sp. XZN143317 | MW826405 |

Table S4 Prevalence of novel astroviruses detected in this study

| **Name** | **Pool** | **Sample source** | **No. of positive samples/ no. of**  **test samples (% positive)** |
| --- | --- | --- | --- |
| Avastrovirus YSN01 | YSNCD | *Montifringilla taczanowskii* | 1/25 (4.0) |
| Avastrovirus YSN02 | YSNCD | Montifringilla taczanowskii | 1/25 (4.0) |
| Avastrovirus XZN01 | XZNCD | sparrow | 1/31 (3.2) |
| Mamastrovirus YSS01 | YSSCD | *Apodemus peninsulae* | 1/50 (2.0) |
| Mamastrovirus YSS02 | YSSCD | *Apodemus peninsulae*, hamster | 2/50 (4.0) |
| Mamastrovirus YSS03 | YSSCD | *Apodemus peninsulae*, *Crocidura* sp. | 2/50 (4.0) |
| Mamastrovirus XZS01 | XZSCD | *Phaiomys leucurus* | 6/41 (14.6) |

Table S5 Amino acid identity (%) of YS19 with RdHEVAc86 and RdHEVAc14.

|  | **ORF1**  **(polyprotein)** | **ORF2**  **(capsid)** | **ORF3** | **ORF4** | **Total** |
| --- | --- | --- | --- | --- | --- |
| **RdHEVAc86** | 86.0 | 91.0 | 71.0 | 88.0 | 85.0 |
| **RdHEVAc14** | 85.0 | 91.0 | 70.0 | 86.0 | 84.0 |


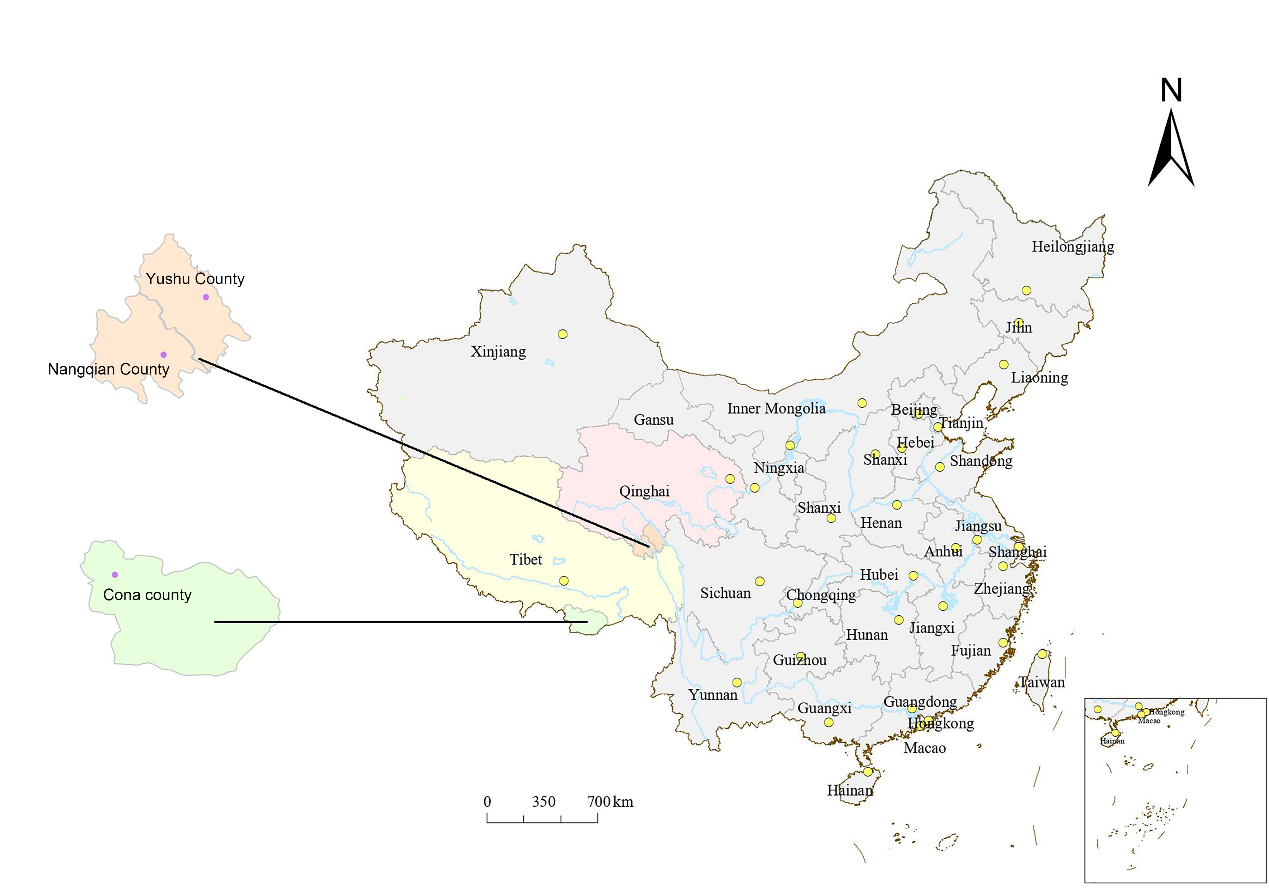


Fig. S1 Map showing location of sampling sites in Tibet and Qinghai Province of China.


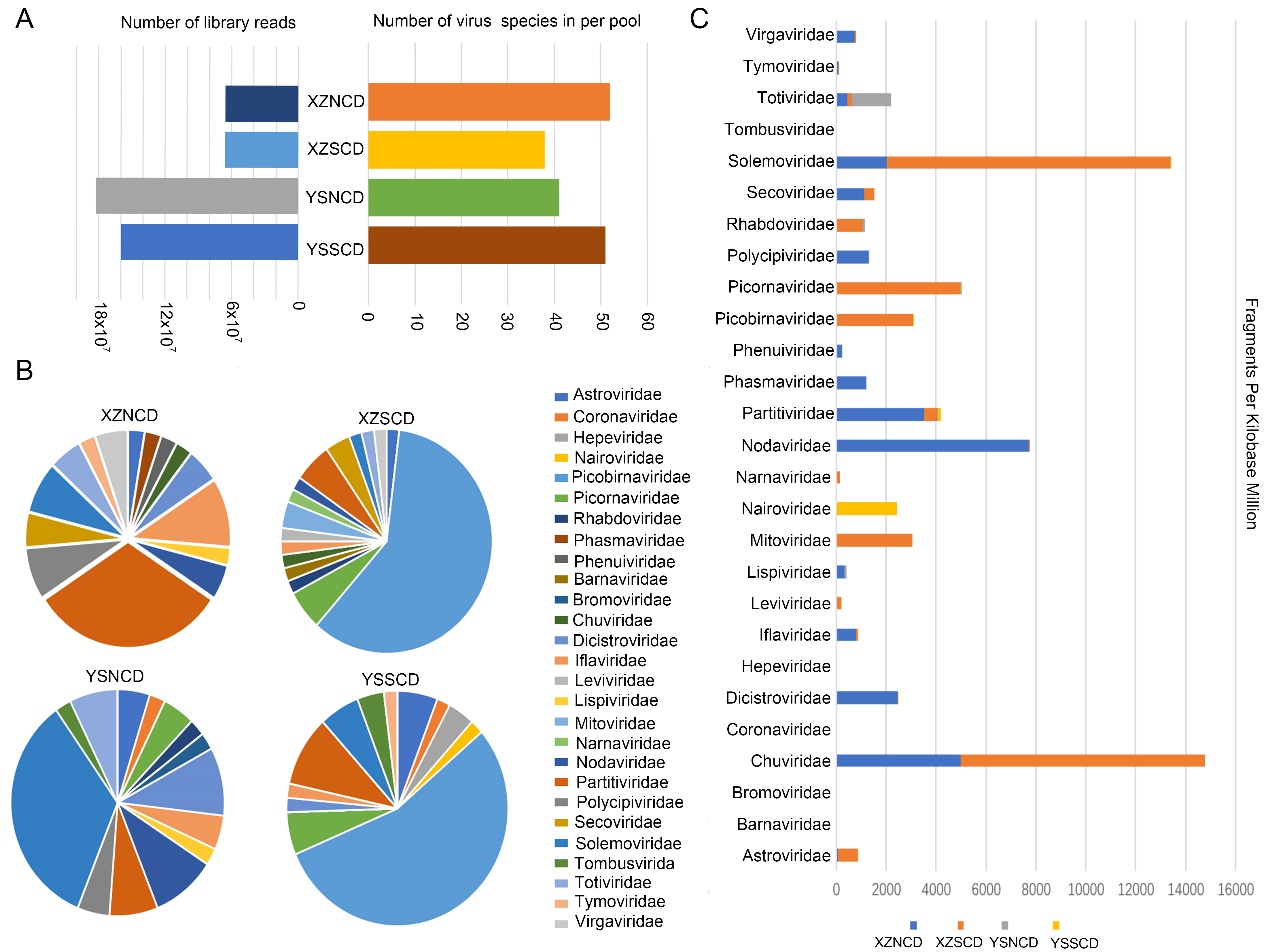


Fig. S2 Overview of the diversity and abundance of RNA viruses. (A) The number of total reads and virus numbers in each library. (B) Distribution of viruses identified in each library. (C) Viral abundance (reads per kilobase million, RPKM) of each library at family level.


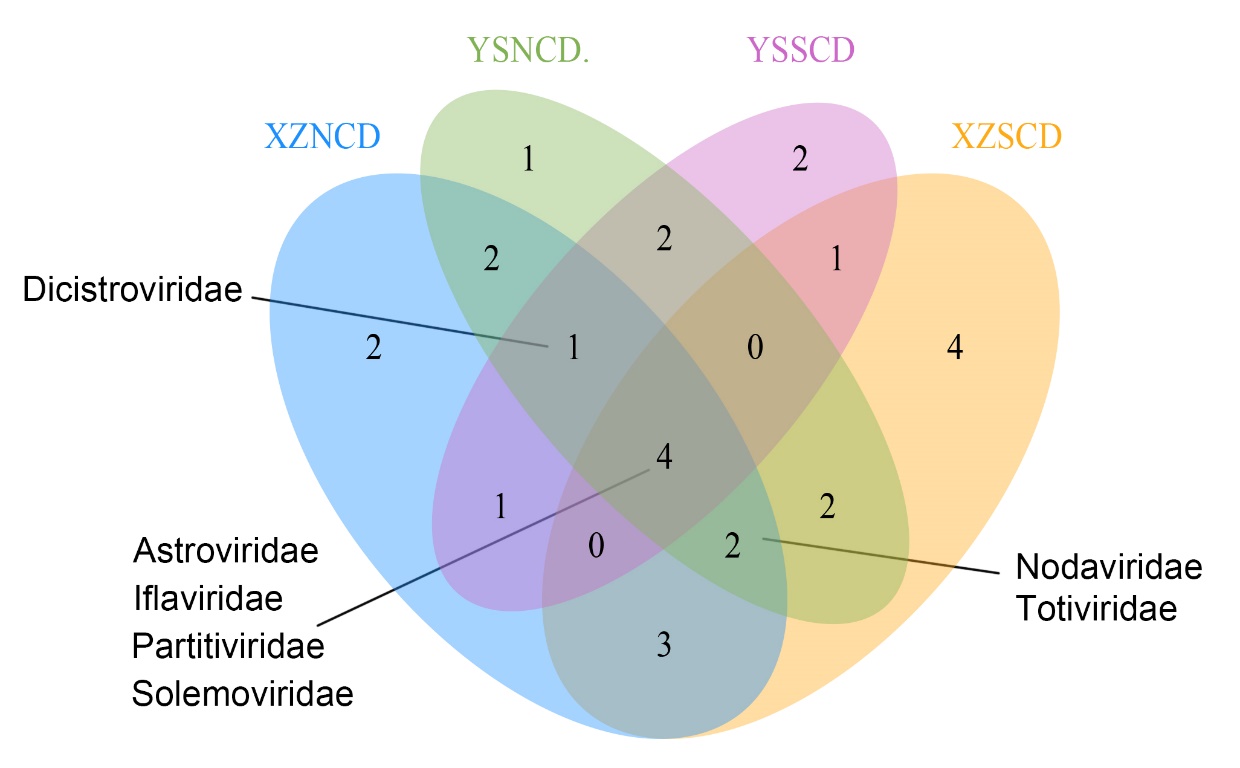


Fig. S3 Overlap of RNA viral families in each library.


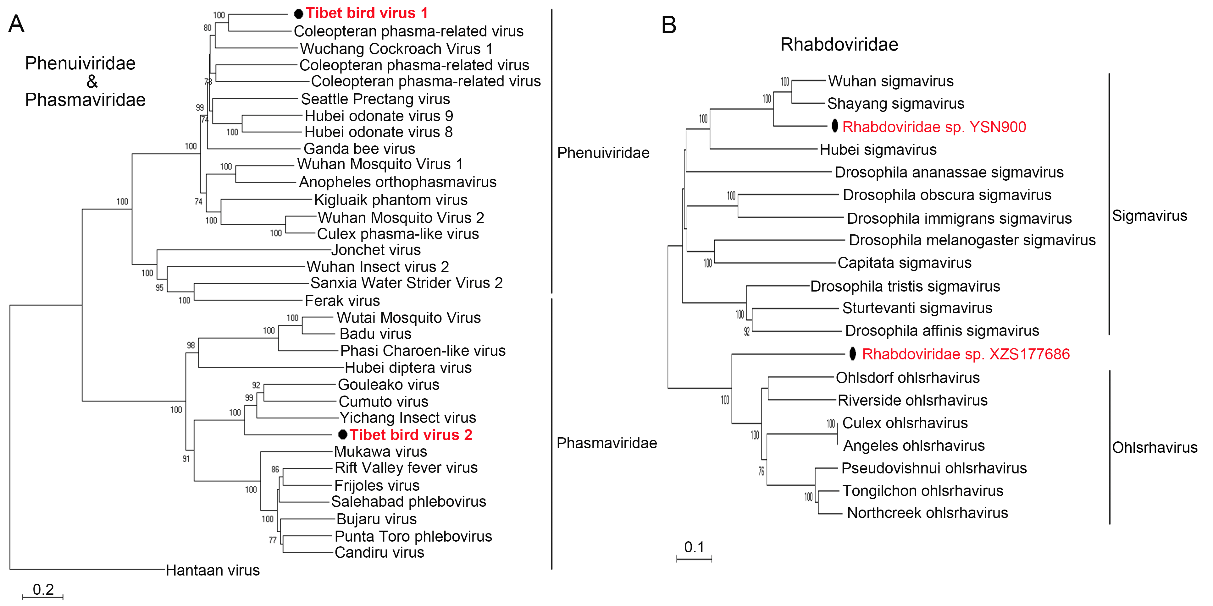


Fig. S4 Phylogenetic relationships of viruses within families *Phasmaviridae* & *Phenuiviridae* (A) and *Rhabdoviridae* (B) based on amino acid sequences of RdRp. Phylogenetic tree was estimated using a maximum likelihood method with 1000 bootstraps. The best-fit substitution model is Dayhoff. Viruses identified here were labeled with solid black circles and red font. Bootstrap values (≥70%) are shown along branches. The name of the virus family is shown above each phylogeny.


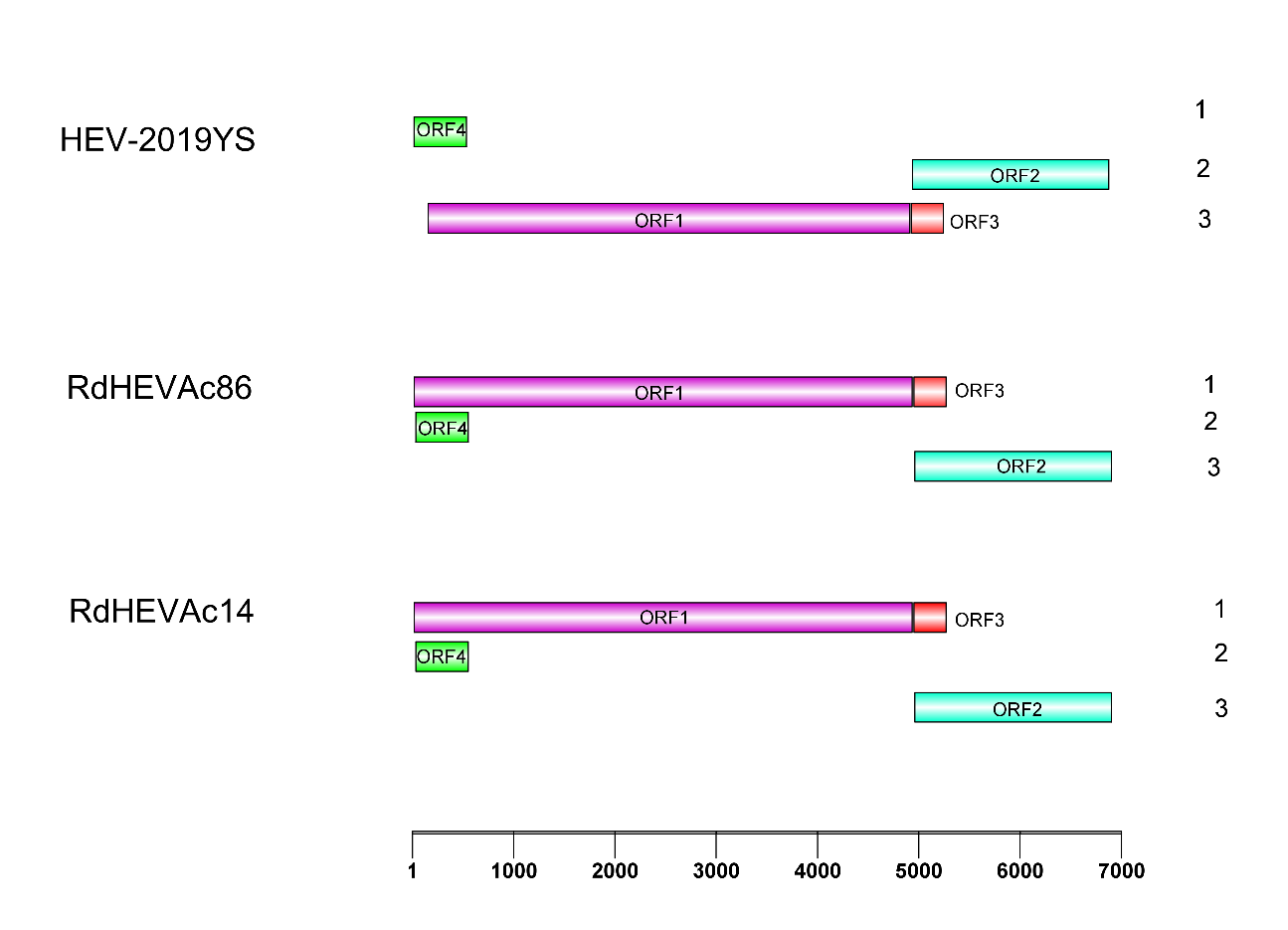


Fig. S5 Genomic characterization of YS19, RdHEVAc86 and RdHEVAc14.


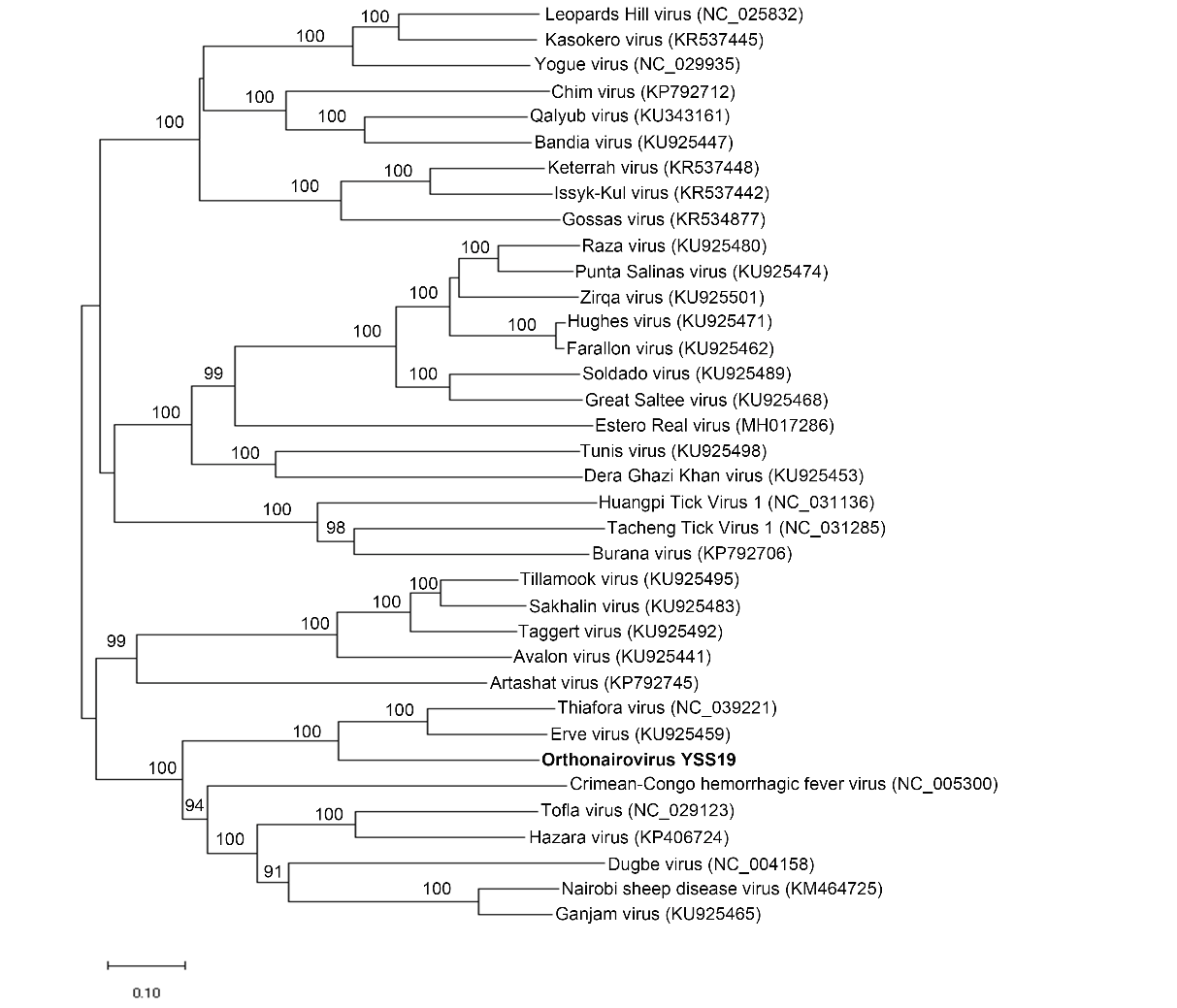


Fig. S6 Phylogenetic analysis based on aa sequences of M segment from all species in genus *Orthonairovirus*. Phylogenetic tree was estimated using a maximum likelihood method with 1000 bootstraps. The best-fit substitution model is Dayhoff. Bootstrap values (≥90%) are showed along branches. Scale bar suggests nucleotide substitutions per site. Viruses from this study are labeled by black bold font.


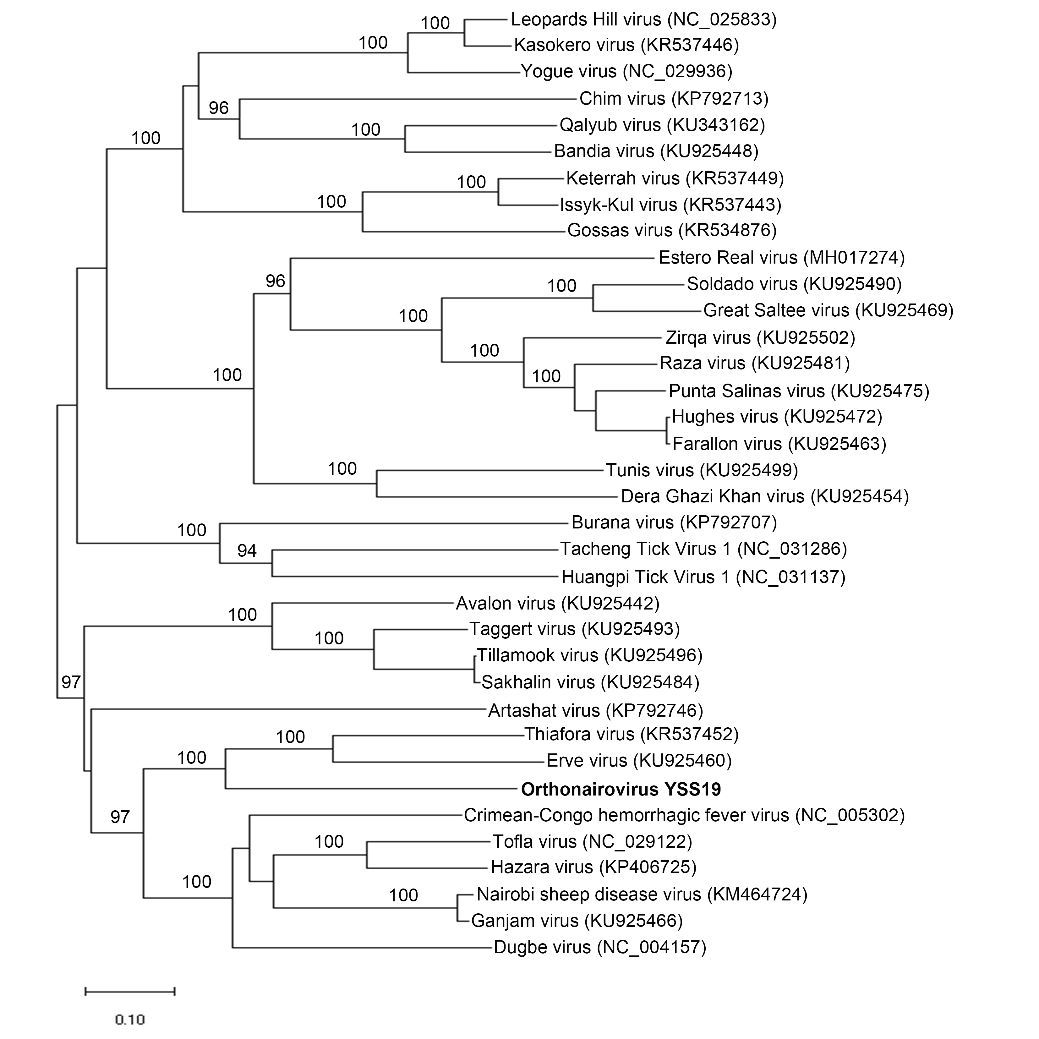


Fig. S7 Phylogenetic analysis based on aa sequences of S segment from all species in genus *Orthonairovirus*. Phylogenetic tree was estimated using a maximum likelihood method with 1000 bootstraps. The best-fit substitution model is Dayhoff. Bootstrap values (≥90%) are showed along branches. Scale bar suggests nucleotide substitutions per site. Viruses from this study are labeled by black bold font.


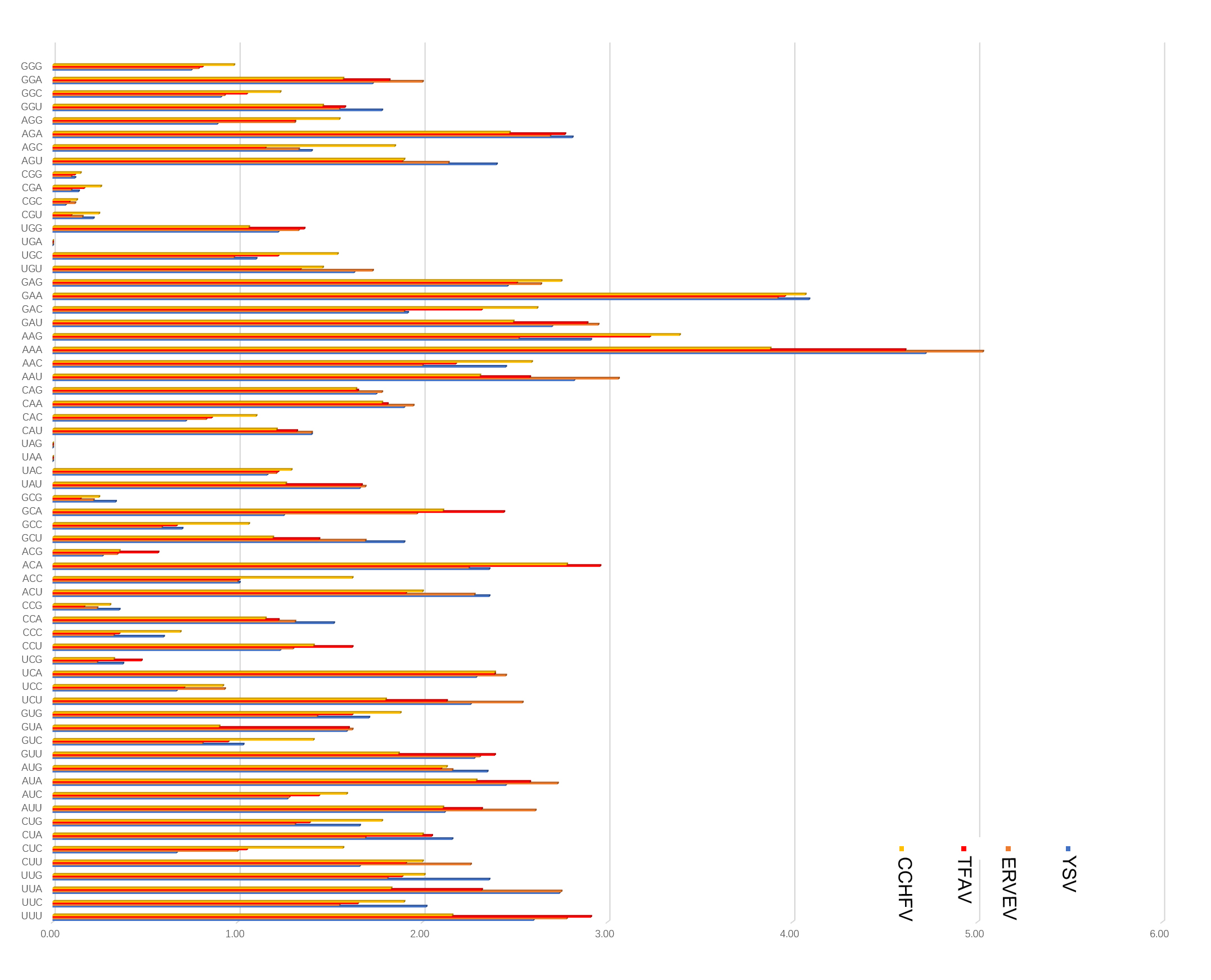
Fig. S8 Codon usages of YSV, ERVEV, TFAV and CCHFV. YSV, YS Orthonairovirus (from this study); ERVEV, Erve virus; TFAV, Thiafora orthonairovirus; CCHFV, Crimean-Congo hemorrhagic fever orthonairovirus.


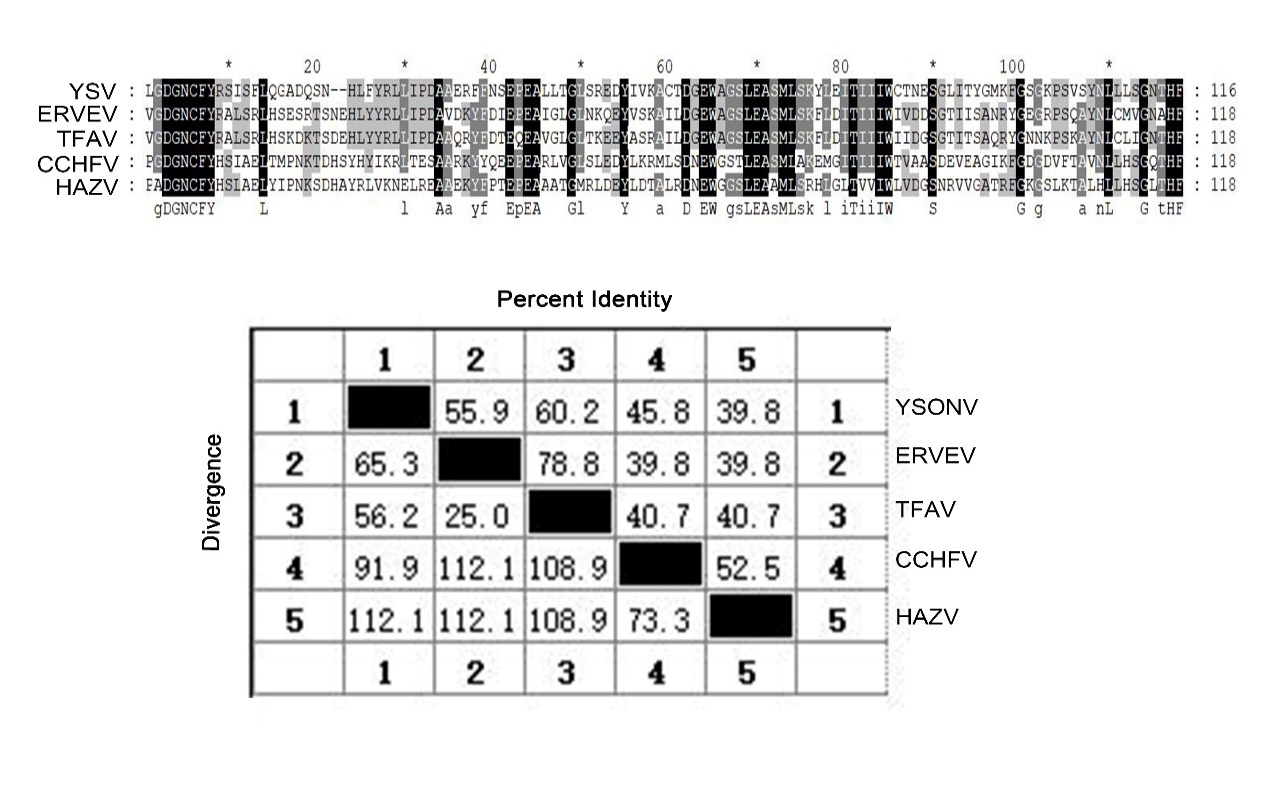
Fig. S9 Sequence pair distances of ovarian tumor (OTU) domain among YSV, ERVEV, TFAV, CCHFV and HAZV. YSV, YS Orthonairovirus (from this study); ERVEV, Erve virus; TFAV, Thiafora orthonairovirus; CCHFV, Crimean-Congo hemorrhagic fever orthonairovirus; HAZV, Hazara orthonairovirus.


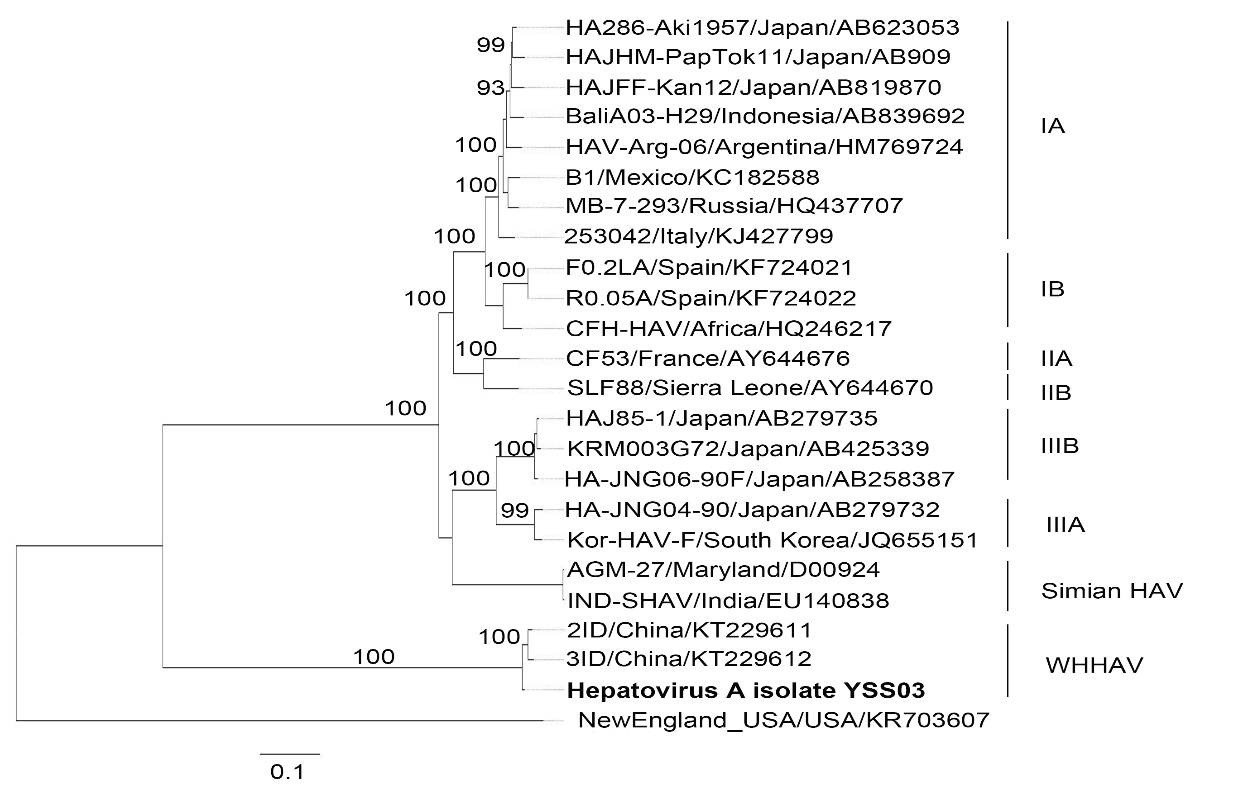
Fig. S10 Phylogenetic analysis based on genomes from genus *Hepatovirus*. Bootstrap values (≥90%) are showed along branches. The phylogenetic tree was estimated using a maximum likelihood method with 1000 bootstraps. The best-fit substitution model is JC. Scale bar suggests nucleotide substitutions per site. Viruses from this study are labeled by black bold font.
